# Do Long-Haul Travel and Jet Lag Affect Athletes’ Physiological, Humoral and Performance Outcomes? A Systematic Narrative Review

**DOI:** 10.3390/sports14030093

**Published:** 2026-03-02

**Authors:** António Benito, Giorjines Boppre, André Lopes, Diogo Cruz, Daniel Moreira-Gonçalves, David Bruce Pyne, Liliana C. Baptista, Rodrigo Zacca

**Affiliations:** 1 Faculty of Sports, Research Center in Physical Activity, Health and Leisure (CIAFEL), University of Porto, Rua lácido da Costa 91, 4200-450 Porto, Portugal; antonio.benito04c@gmail.com (A.B.); up202310129@edu.fade.up.pt (D.C.); danielmgon@fade.up.pt (D.M.-G.); 2 Laboratory for Integrative and Translational Research in Population Health (ITR), Rua das Taipas 135, 4050-600 Porto, Portugal; 3 Institute for Molecular Sports and Rehabilitation Medicine, Paracelsus Medical University, 5020 Salzburg, Austria; giorjines.boppre@pmu.ac.at; 4 Research Institute for Sport and Exercise (UCRISE), University of Canberra, Canberra 2617, Australia; david.pyne@canberra.edu.au; 5 Faculty of Sport Sciences and Physical Education, Interdisciplinary Center for the Study of Human Performance, University of Coimbra, 3040-248 Coimbra, Portugal; lbaptista@fcdef.uc.pt; 6 Nucleus of Research in Human Motricity Sciences, Universidad Adventista de Chile, Chillán 3780000, Chile

**Keywords:** long-haul travel, athletes, travel fatigue, sports performance, circadian rhythms, autonomic nervous system, athletic readiness, hemodynamic response

## Abstract

**Background**: Long-haul travel and jet lag can disrupt athletes’ circadian, physiological, and performance systems, potentially impairing competition outcomes. This review aimed to study the effects of long-haul travel on athletes’ health and performance, differentiate travel fatigue from jet lag, and review mitigation strategies. **Methods**: A systematic narrative review was conducted following PRISMA 2020 guidelines. PubMed, Scopus, and Web of Science were searched for studies on jet lag, travel fatigue, and long-haul travel in athletes. Eligibility included studies reporting physiological, hemodynamic, or performance outcomes in athletes of any level and sex. Data were extracted on travel characteristics, interventions, physiological and performance markers, and risk of bias. **Results**: Overall, 284 records were identified, with 89 studies included. Travel directions were equally distributed between eastward and westward journeys, crossing 1–12 time zones. Interventions to mitigate travel effects were reported in 17 studies, primarily melatonin, caffeine, and light exposure. Common physiological changes included sleep disturbances (*n* = 36), body temperature alterations (*n* = 18), blood pressure changes, hormonal shifts (*n* = 9), heart rate variability (*n* = 4), and immune alterations (*n* = 4). Travel effects comprised fatigue (*n* = 25), sleep changes (*n* = 21), decreased physical performance (*n* = 18), mood changes (*n* = 15), and cognitive impairments (*n* = 9). Physical performance outcomes included anaerobic power (*n* = 18), strength (*n* = 14), velocity (*n* = 12), aerobic capacity (*n* = 10), coordination (*n* = 8), and reaction time (*n* = 7). Risk of bias was low in 49%, moderate in 17%, and high in 34% of studies. **Conclusions**: Long-haul travel negatively affects multiple physiological and performance domains in athletes, including sleep, hormonal balance, autonomic function, and physical performance. The magnitude of these effects seems to be influenced by travel direction, number of time zones crossed, and individual susceptibility. Eastward travel is generally associated with stronger circadian disruption and impaired aerobic capacity, coordination, and technical performance, whereas westward travel often induces greater fatigue and adversely affects team-sport outcomes. Monitoring key markers such as heart rate variability, sleep, and cortisol, combined with personalized strategies including circadian management, sleep hygiene, nutrition, recovery interventions, and training load adjustments, is essential to mitigate travel-related impairments and optimize performance.

## 1. Introduction

The modern athlete, whether elite or recreational, spends a substantial portion of the competitive season traveling for training and competition [1]. This is a direct consequence of the increasingly globalized nature of sport and may involve long-haul intercontinental flights or, more commonly, frequent short-distance travel. Such travel exposes athletes to two related but distinct challenges: travel fatigue and jet lag [2]. These conditions share several symptoms and, due to limited awareness, athletes often incorrectly attribute all travel-related effects exclusively to jet lag [3,4,5]. Although these conditions share several symptoms and are often conflated in practice, they represent different phenomena. However, travel fatigue and jet lag are distinct phenomena that may co-occur when traveling eastward or westward [6]. Travel fatigue is a condition arising from the inherent stressors of the journey itself, such as airport procedures, travel logistics, sleep loss, dehydration, and cramped seating, and can occur regardless of the distance or number of time zones crossed. Jet lag, however, results from a misalignment between the body’s internal clock and the destination’s local time and typically occurs only when crossing 3 or more time zones [6].

Regardless of flight direction or duration, any form of travel can induce fatigue [7,8,9]. These symptoms may occur acutely after a single journey or develop chronically as a cumulative consequence of repeated travel throughout a competitive season [6]. Travel fatigue can occur independently of the number of time zones crossed and results from the inherent stressors associated with travel itself. It often begins prior to departure due to factors such as prolonged airport procedures, flight delays, and pre-competition anxiety. During the flight, contributing factors include cramped seating position, reduced physical activity, mild hypoxia, and dehydration caused by low cabin humidity. Upon arrival, additional stressors such as unfamiliar environments and cultural differences may further exacerbate travel fatigue symptoms [4].

**Figure 1:**
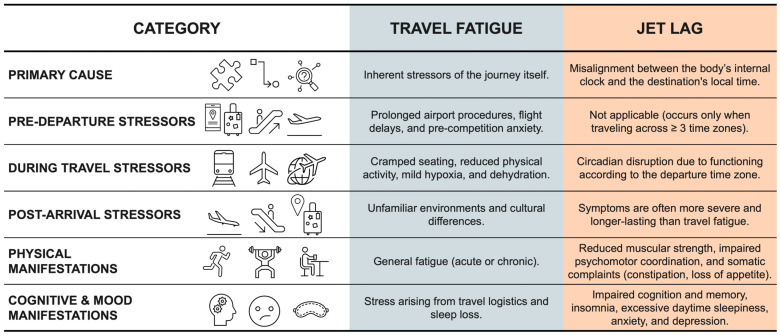
Stressors and manifestations of travel fatigue and jet lag.

In contrast, jet lag results specifically from a circadian misalignment between the body’s internal clock and the local time at the destination. Jet lag shares symptoms with travel fatigue; however, these are often more severe and longer lasting [6,10,11]. Both travel direction and duration have direct implications for symptom manifestation, but jet lag typically occurs when journeys involve crossing 3 or more time zones [6]. Depending on the timing of training or competition, this circadian disruption can directly impair athletic performance [6]. Both physical and cognitive performance may be adversely affected by jet lag, leading to symptoms such as insomnia and/or excessive daytime sleepiness, fatigue, reduced muscular strength and psychomotor coordination, impaired cognition and memory, anxiety, depression, and somatic complaints including constipation and loss of appetite [12]. These manifestations occur because the body continues to function according to the circadian rhythm of the departure time zone [13]. Despite frequent references to performance impairment, there remains a gap in the literature regarding which specific domains are most affected. Figure 1 summarizes the practical stressors and manifestations of travel fatigue and jet lag described in the current literature.

Westward travel lengthens the day, resulting in a delay of the biological clock, whereas eastward travel shortens the day, leading to an advance of the biological clock [14]. Consequently, the time required to resynchronize the internal circadian rhythm with the local time varies according to the direction of travel. Westward travel generally requires approximately 0.5 days of adjustment per hour of time difference, eastward travel typically requires about 1.5 days per hour of difference [11,12,14]. It is also important to note that eastward travel tends to produce more pronounced jet lag symptoms, as the human circadian system is naturally better adapted to extending the day than shortening it [14,15].

Circadian rhythms, from the Latin *circa dies* (about a day), govern the daily rhythm of human physiology and performance [15,16]. Most physiological functions exhibit circadian rhythmicity, characterized by 24 h oscillations with maximum (acrophase) and minimum (nadir) values occurring at specific times of day [8,15]. Circadian rhythms are expressed as oscillations in physiological systems, including body temperature, heart rate, and hormonal levels such as cortisol. These parameters respond to both internal (e.g., neurotransmitters or metabolic substrates) and external (e.g., environmental factors, drugs, food) stimuli [8,15,17]. In humans, the primary synchronizer of circadian rhythms is the light–dark cycle [4,8,15,18].

The circadian rhythm is regulated by the suprachiasmatic nucleus (SCN), located in the hypothalamus, which is considered the master biological clock. The SCN regulates, among other functions, the production of melatonin by the pineal gland and interacts with neurotransmitters such as serotonin, which indirectly participates in sleep regulation and serves as a precursor to melatonin. Through these mechanisms, the body transitions from daytime alertness to nighttime drowsiness [4]. The SCN sends signals to different regions of the hypothalamus, influencing processes such as thermoregulation, hormone secretion, feeding and sleeping, thereby highlighting its central role in coordinating the organism’s physiological functions [8].

External timing cues, known as *zeitgebers* (German for “time-givers”), synchronize the body’s internal clock network with the external environment. In humans, light is the most potent zeitgeber. The SCN receives photic input from the retina and uses it to reset subordinate clocks throughout the brain and peripheral tissues through direct neural projections, humoral signaling, or by regulating behavioral rhythms such as the sleep–wake and feeding–fasting cycles [19]. Restricting food intake to the normal rest phase (nighttime in humans, daytime in nocturnal rodents) can uncouple peripheral clocks from the SCN pacemaker, with glucocorticoids playing a key role. This internal desynchronization is characteristic of shift work and may contribute to an increased risk of obesity, type 2 diabetes, cardiovascular disease, and mood disorders [19]. In fact, cellular clocks assist to regulate blood pressure, heart rate, and endothelial function [19]. When these clocks are disrupted, the risk of conditions such as heart disease, heart failure, myocardial infarction, and arrhythmias increases.

**Figure 2:**
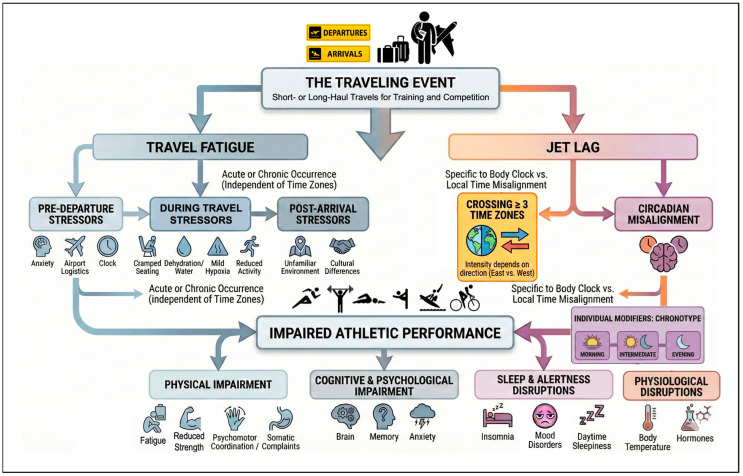
Conceptual framework illustrating the pathways linking travel to athletic performance. Travel induces travel fatigue (independent of time zones) and jet lag (dependent on time zones crossed), distinct challenges that disrupt the central circadian rhythm (SCN). This physiological disruption, modulated by individual chronotype, negatively affects sleep, hormone regulation, and cognition, leading to specific consequences that compromise athletic performance and training adaptation.

Humans exhibit interindividual differences in the timing of behavioral patterns, including social activities, daytime routines, and sleep patterns [20]. These differences are commonly described along a chronotype continuum, ranging from morning types to evening types, with intermediate types occupying the midpoint [20]. Morning types display phase-advanced tendencies, showing a preference for early awakening and morning activity, and often experience difficulty remaining awake beyond their habitual bedtime [20]. In contrast, evening types are phase-delayed, preferring later bedtimes and experiencing difficulty waking early [20]. In the general adult population (ages 19–31), chronotype distribution indicates that most individuals are intermediate types (70%), with smaller proportions classified as morning (14%) or evening types (16%) [21]. In elite sports, athletes’ sleep–wake patterns are strongly influenced by training and competition schedules [22]. It is important to underline that the knowledge produced for the benefit of those who often operate at the extreme boundaries of human capacity, such as elite athletes, could be applied to improve the quality of life of the general population. Indeed, there are various examples of this transfer from elite sport to general population. Optimal training adaptation and performance are thought to depend on the alignment between an athlete’s chronotype and their training schedule [23]. Accordingly, athletes engaged in predominantly morning-based sports tend to be morning types, whereas those participating in evening-based sports tend to be evening types [20] (Figure 2).

Thus, the primary aims of this systematic narrative review were: (i) to explore the importance of circadian rhythms for athletes’ health and performance, (ii) to distinguish between the terms jet lag and travel fatigue, (iii) to examine how athletes and teams prepare for long-haul travel before competitive events, (iv) to identify the markers used to assess athletes’ readiness, and (v) to investigate whether long-haul travel induces physiological, hemodynamic, or performance-related changes.

## 2. Materials and Methods

### 2.1. Eligibility

While adhering to the rigorous search standards of a traditional systematic review, we adopted a “systematic narrative review” design, which differs from a traditional systematic review by substituting statistical quantification with a narrative synthesis to effectively integrate data from diverse methodologies. The elements of this systematic narrative review methodology that were applied and those which were not are reported at the International Prospective Register of Systematic Reviews (PROSPERO; registration number: CRD42025630974). This systematic narrative review was conducted and reported in accordance with the PRISMA 2020 (Preferred Reporting Items for Systematic Reviews and Meta-Analyses) guidelines (https://www.prisma-statement.org/ accessed on 3 December 2024) [24] (see the PRISMA 2020 checklist as Supplementary File S1). Three databases were assessed for the research: PubMed, Scopus and Web of Science (search dates: until January 2025). The search string and Boolean operators used were: ((“jet lag”) OR (“travel fatigue”) OR (“long travel”) OR (“transmeridian travel”) AND (“performance”) AND (“athletes”). Participants, interventions, comparators, outcomes, and study design (PICOS) were defined as follows:

- Participants—Athletes of all levels, ages, and both sexes (male and female);
- Interventions—Jet lag and long-haul travel. Long-haul travel refers to journeys that typically exceed 4000 km and often involve crossing multiple time zones or continental boundaries. While long-haul travel offers significant economic and cultural benefits, it may also pose specific challenges, including health and performance-related impacts;
- Comparators—Athletes who are frequently exposed to long-haul travel for training and/or competition;
- Outcomes—The primary outcomes in this review include humoral alterations, gastrointestinal disturbances, circadian rhythm disruptions, psychophysiological responses, hemodynamic changes, and variations in blood biomarkers. Additionally, secondary outcomes encompass physical fitness and performance-related indicators such as cardiorespiratory fitness, body composition, bone health, and various measures of muscular strength (isometric, dynamic, explosive, and reactive), as well as flexibility, agility and speed;
- Study Design—The review did not impose restrictions on study design, and all types of studies were deemed eligible for inclusion.

To ensure methodological rigor, the following systematic elements were implemented: prior protocol registration in PROSPERO, adherence to PRISMA 2020 reporting guidelines, and predefined eligibility criteria based on the PICOS framework. The process included a comprehensive multi-database search, dual independent screening and selection, structured data extraction using a predefined template, and independent risk of bias assessment using the JBI Critical Appraisal Tools.

### 2.2. Study Selection

After defining the Boolean operators and keywords for the literature search (Table 1), all retrieved references were imported into an EndNote 21 database (version 21.5); https://endnote.com, London, UK. Automatic duplicate removal tool was performed, followed by manual verification to identify and remove any remaining duplicates. Subsequently, two authors independently screened the titles and abstracts to determine eligibility for full-text review or exclusion. Disagreements between the two reviewers were resolved by consultation with a third author, who made the final decision. Full-text articles selected for inclusion were then read in detail, and a third stage of selection was applied: studies whose topic was not related to the aim of the review were excluded at this stage. The full-text assessment was performed independently by the same two reviewers using predefined eligibility criteria, with disagreements resolved through consultation with a third reviewer following the same procedure.

### 2.3. Data Extraction

From the eligible studies that were included in this review, data that was extracted from the studies were: publication details (first author, study tittle, year of publication), study design, participants (sex, sample size, competitive level), travel details (direction, number of time zones crossed), supplementation (exogenous strategies to mitigate travel effects), main results and conclusions.

### 2.4. Risk of Bias Assessment

The methodological quality of the included studies was assessed independently by two reviewers using the appropriate Joanna Briggs Institute (JBI) Critical Appraisal Tools, according to the specific study design. Each criterion within the tool was rated as “yes,” “no,” “unclear,” or “not applicable.” The results of the risk of bias assessment were used to inform the interpretation of findings but did not serve as exclusion criteria for studies. The following thresholds were established: low risk of bias (“yes” scores ≥ 70%), moderate (50 ≤ “yes” scores < 70%), and high (“yes” scores < 50%). Specifically, the JBI Critical Appraisal Checklists for Case Series, Case Reports, Quasi-Experimental Studies, Systematic Reviews, Cohort Studies, Randomized Controlled Trials, Qualitative Research, Expert Opinion, and Analytical Cross-Sectional Studies were applied according to the specific design of each included study.

## 3. Results

**Figure 3:**
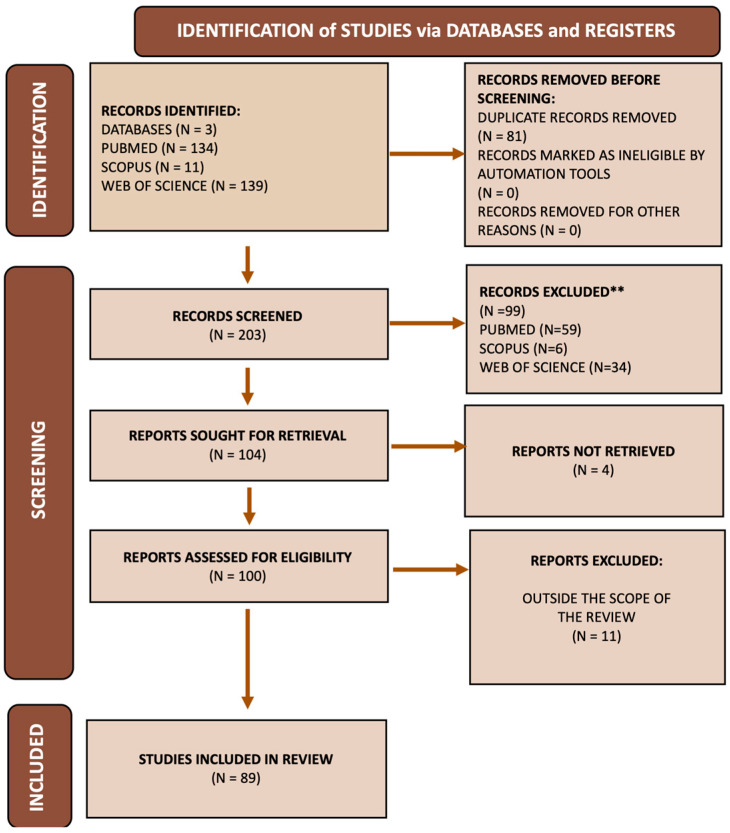
Flow chart.

Overall, a total of 284 records were identified using the search strategy as described above. Of the 100 studies retained for full-text screening, 11 did not meet inclusion criteria and were removed. 89 studies were therefore deemed eligible for inclusion in the review (Figure 3). Table 2 shows the 89 studies included in the review.

The 89 studies were published between 1985 and 2024 and included the sports of soccer (*n* = 21); Rugby (*n* = 12); American Football (*n* = 8); Basketball (*n* = 8); Swimming (*n* = 7); Athletics (*n* = 7); Cycling (*n* = 6); Skeleton (*n* = 4); Super Rugby (*n* = 4); Tennis (*n* = 3); Track and field (*n* = 3); Rowing (*n* = 3); Triathlon (*n* = 3); Badminton (*n* = 2); Golf (*n* = 2); Ice Hockey (*n* = 2); Volleyball (*n* = 2); Gymnastics (*n* = 2); Boxing (*n* = 2); Sailing (*n* = 2); Cricket (*n* = 2); Hockey (*n* = 2); High Jump/Long Jump (*n* = 1); Baseball (*n* = 1); Wheelchair basketball (*n* = 1); Netball (*n* = 1); Speed skaters (*n* = 1); Biathlon (*n* = 1); Kite surfers (*n* = 1); Wrestling (*n* = 1); Clay target and shooting (*n* = 1); Diving (*n* = 1). Regarding study design, the included studies comprised narrative reviews (*n* = 21), critical reviews (*n* = 2), and systematic reviews (*n* = 2). Primary research designs included observational studies (*n* = 37) and cohort studies (*n* = 3). Experimental designs consisted of experimental studies (*n* = 3), quasi-experimental studies (*n* = 4), and randomized control trials (*n* = 2). Additional study types included case studies (*n* = 7), case reports (*n* = 1), qualitative studies (*n* = 2), commentaries (*n* = 3), a position statement (*n* = 1) and a cross-sectional survey (*n* = 1). It is noteworthy that although significant impairments are often emphasized, several included studies yielded non-significant findings or null effects across various performance parameters. Therefore, the evidence should be interpreted with caution, acknowledging that the absence of a statistically significant effect in certain cohorts is as informative as its presence (See Table 2).

### 3.1. Travel Details

Of the 89 studies included in this review, approximately 33 did not report any travel details (e.g., duration, direction, time zones crossed). 49 studies reported the direction of travel, with 23 studies for eastward and 23 studies for westward journeys, followed by northeast (2 studies). 1 study reported travels across all North America. Regarding time zones, 31 studies reported the number of time zones crossed. Overall, participants crossed between 1 and 12 time zones. The most frequently crossed time zones were: 8 time zones (*n* = 6), 5 time zones (*n* = 5), 7 time zones (*n* = 4), 12 time zones (*n* = 3), 9 time zones (*n* = 3), 4 time zones (*n* = 2), 10 time zones (*n* = 2), 1 time zone (*n* = 1), 2 time zones (*n* = 1), 3 time zones (*n* = 1), 6 time zones (*n* = 1) and 11 time zones (*n* = 1) (See Table 2).

### 3.2. Interventions to Mitigate Travel Effects

Of the 89 studies included in this review, 72 did not report any intervention to mitigate travel effects. Among those that did, melatonin was cited in 16 studies, caffeine in 6 studies and light exposure in 3 studies. Other reported interventions included physical exercise, probiotics and ramelteon being cited in 1 study (See Table 2).

### 3.3. Physiological and Hemodynamic Markers

In terms of physiological and hemodynamic changes, 36 studies cited sleep changes. Among these, 15 studies used objective measures (e.g., actigraphy, polysomnography), of which 10 also included subjective self-reports. Meanwhile, 24 studies relied on subjective measures (e.g., sleep diaries, questionnaires), with 10 of these also reporting objective sleep outcomes. Alongside sleep disturbances, other physiological markers were consistently reported. These included alterations in body temperature (*n* = 18), blood pressure, hormonal changes (*n* = 9), heart rate variability (*n* = 4), and immune system (*n* = 4). It should be noted, however, that HRV outcomes were reported in only 4 studies, underscoring the scarcity of evidence and necessitating a cautious interpretation of these results. (See Table 2).

Regarding immune and inflammatory outcomes, the findings demonstrate substantial heterogeneity rather than a consistent trend. While long-haul travel is associated with immune system disruption in some studies (*n* = 4), the specific markers and the direction of these changes vary significantly across the literature. This inconsistency suggests that immune responses may be more sensitive to individual variability, cabin environment, or the specific stressors of the travel itinerary rather than following a uniform temporal pattern of disruption.

Temporal analysis of these markers reveals that physiological disruption is immediate, with core body temperature rhythms markedly altered as early as Day 1 post-travel, regardless of flight direction. Regarding performance and psychophysical state, the most acute impact occurs between Days 1 and 2, a period characterized by peaks in fatigue, confusion, and reductions in anaerobic power. The recovery trajectory is variable and depends on the stabilization of the sleep–wake cycle. While improvements in mood and strength may occur within a few days, the full normalization of hemodynamic variables and core temperature is slower, typically extending for about 1 week, although in individual cases, the recovery period may range from 3 to 11 days.

### 3.4. Travel Effects

Regarding the effects imposed by travel, fatigue was cited in 25 studies, sleep changes (e.g., insomnia, bad sleep quality) in 21 studies, decreased physical performance (e.g., strength, power, coordination, velocity) in 18 studies, mood changes (e.g., irritability, confusion, demotivation) in 15 studies, cognitive problems (e.g., attention, reaction time, concentration) in 9 studies (See Table 2).

### 3.5. Physical Performance Markers

In the 89 studies included, 69 reported physical performance outcomes. Anaerobic power (e.g., sprints, jumps) was cited in 18 studies. Strength (e.g., grip, isometric) was reported in 14 studies. Velocity was cited in 12 studies. Aerobic power and capacity were cited in 10 studies. Coordination was cited in 8 studies. Reaction time was reported in 7 studies.

### 3.6. Risk of Bias

Of all studies included in this systematic narrative review, 49% of studies showed low risk of bias, 17% of studies showed moderate risk of bias, and 34% of studies showed high risk of bias. The average score of all studies was 61%, which indicates an overall rating of moderate risk of bias (Table 3).

## 4. Discussion

Long-haul travel disrupts multiple physiological and performance systems in athletes. It is important to distinguish between the acute effects of travel fatigue (driven by non-circadian stressors such as sleep deprivation, dehydration, and logistical constraints) and the persistent physiological shifts caused by circadian misalignment (jet lag). While travel fatigue typically resolves with rest and hydration, the effects of circadian misalignment are influenced by travel direction, the number of time zones crossed, and individual variability. Despite their distinct etiologies, these stressors often coexist in elite athletic travel. As illustrated in the conceptual framework of Figure 2, these factors trigger a cascade of physiological disruptions. Consequently, their cumulative impact impairs aerobic and anaerobic performance, strength, velocity, coordination, and sport-specific skills, although some outcomes differ across studies. However, it is essential to acknowledge that associations, for example, between travel direction and match outcomes in team sports, may be influenced by several confounding factors, such as team quality, scheduling, tactics, and opponent level. Therefore, while a potential causal link is suggested, these findings should be interpreted with caution, as they may not solely reflect the direct physiological effects of jet lag. Targeted recovery, differentiating between acute fatigue management and circadian resynchronization, is essential to preserve performance. While some physiological disruptions are well-documented, other mechanistic explanations, such as specific hormonal imbalances, require further empirical validation in the context of elite athletic travel. Similarly, supplementation was not the focus of most studies, but repeated mentions of caffeine suggest potential benefits, though evidence remains inconclusive. Jet lag may be less pronounced when travel coincides with major competitions or meaningful events [98]. These changes may contribute to cardiovascular rhythm disturbances during and after long-haul travel.

### 4.1. Physiological and Hemodynamic Markers

Circadian rhythms that persist under constant conditions originate within the body, indicating an endogenous component [15]. Environmental and behavioral factors such as sleep, activity, and light exposure modulate these rhythms, representing the exogenous component [8]. During the day, body temperature is elevated due to the combined effect of the internal clock and alerting influences of environmental factors and activity. At night, the internal clock, environmental cues, and reduced activity all contribute to lower body temperature. This interaction illustrates the multifactorial regulation of physiological variables under travel conditions.

#### 4.1.1. Body Temperature

Irrespective of flight direction, core body temperature rhythms were markedly disrupted on day 1. Eastward travel produced a phase advance, whereas westward travel induced a phase delay [29]. Core body temperature was strongly coupled to the sleep–wake cycle, and temperature regulation appeared to recover only after this cycle stabilized [30,31]. Recovery from disruption ranged from 3 to 11 days, although some individuals recovered by day 3 [35,77]. This suggests a potential causal link, although the underlying biological drivers remain to be fully elucidated. Restricted or fragmented sleep further impairs temperature regulation [4]. Transmeridian travel disrupts core temperature rhythms independent of direction, with recovery generally occurring within 1 week [29].

#### 4.1.2. Blood Pressure

In German Olympic gymnasts, westward travel increased peak systolic and diastolic values, whereas eastward travel decreased them [29]. These direction-dependent effects are influenced by altitude, cabin pressure, vibration, and fatigue [8]. Westward flights tend to raise blood pressure, whereas eastward flights tend to lower it. These changes may contribute to cardiovascular rhythm disturbances during and after long-haul travel.

#### 4.1.3. Sleep

Sleep is consistently disrupted after transmeridian travel. In professional soccer players, total sleep duration remained largely unchanged, but awakenings increased on days 1 and 3, and sleep latency decreased on days 2, 6, and 8 after westward travel [28]. Timed exercise, bright light exposure, and melatonin accelerate circadian re-synchronization [28,37,64,99]. Eastward travel delayed bedtime and wake times, increasing total sleep duration, whereas westward travel often reduced sleep quality [9,99]. Interventions such as timed exercise, light exposure, and melatonin mitigate these disturbances.

#### 4.1.4. Cortisol

Cortisol is a key stress hormone and a central marker of the hypothalamic–pituitary–adrenal (HPA) axis [100]. However, the literature is inconsistent regarding the effects of travel on cortisol secretion. After eastward travel, cortisol levels increased [35,61], whereas Bullock et al. reported a decrease [33]. In contrast, Stevens et al. found no change in cortisol levels after travel [54]. When disruptions occurred, 7 to 11 days were required for cortisol levels to return to baseline in both eastward and westward directions [29,33,77]. Such changes can impair physiological processes related to performance, recovery, and immune function. The endocrine response is variable, emphasizing the need for personalized strategies to mitigate travel-related effects on cortisol and the endocrine system [34]. Therefore, monitoring athletes’ cortisol after transmeridian travel is essential, as disruptions can affect performance, recovery, and immunity. Interventions may include timed sleep and light exposure, scheduled exercise, appropriate nutrition and hydration (including easily digestible foods), stress-management techniques, and, where appropriate, supervised melatonin to support endocrine recovery.

#### 4.1.5. Heart Rate Variability (HRV)

Heart rate variability (HRV) is a robust indicator of autonomic function and the balance between sympathetic and parasympathetic activity [101]. Its sensitivity to physiological changes makes HRV a relevant marker for the effects of long-haul travel [36]. The literature is inconsistent regarding HRV responses. HRV declined after transmeridian travel crossing more than 3 time zones [27,36,78], but values typically returned to baseline at a rate of 1 day per time zone crossed [36]. After crossing 5 time zones, HRV remained impaired on days 4 and 5, with progressive recovery until day 13, suggesting that the autonomic system requires several days to reestablish stability following circadian disruption [36]. HRV is also influenced by prior-day training loads, indicating an interaction between environmental factors (e.g., temperature, humidity) and training-related physiological stress. More recently, a temporary decrease in LnRMSSD (natural logarithm of the root mean square of successive R-R interval differences) was observed, more pronounced in non-starters, suggesting that exposure to competition and game load modulates autonomic responses to jet lag [78]. Therefore, HRV is a sensitive, non-invasive marker of autonomic disruption after long-haul travel, with recovery timelines proportional to the number of time zones crossed. Monitoring HRV can guide individualized training-load adjustments and recovery strategies, optimizing cardiovascular adaptations, mitigating the physiological impact of jet lag, and supporting athletes’ performance and readiness.

### 4.2. Physical Performance Markers

Physical performance outcomes are heterogeneous. The higher the performance level, the smaller the differences between athletes and the narrower the gap between winners and losers. In elite competitions, success often hinges on minute details. As professional athletes constantly seek a competitive edge, manipulating circadian rhythms may confer an advantage [17]. Most performance-related parameters (grip strength, anaerobic and aerobic power, hormonal secretion, and self-selected work rate) are closely linked to the body temperature rhythm, which peaks in the late afternoon (around 18:00) [8,17]. Notably, a disproportionate number of world records have been set during this period [17,18].

#### 4.2.1. Anaerobic Power

Effects of long-haul travel on anaerobic power are inconsistent across studies. In athletes and healthy, physically trained individuals, long travel can reduce muscle performance, vertical jump height, and maximal acyclic power, regardless of travel direction, while simpler tasks typically recover more quickly [4,25,38,50,77]. Maximal and intermittent sprint performance may be impaired for up to 9 h post-travel [50]. In male professional soccer players, high-intensity actions, such as sprint distance and high-intensity running during games, were negatively affected after travel [68]. However, in skeleton athletes after eastward travel crossing 4 time zones, no differences were observed in post-travel 30 m sprint time [33]. Conversely, in highly trained rowers traveling westward across 9 time zones, the propulsive phase and eccentric velocity during loaded countermovement jumps (LCMJ) improved, resulting in higher mean power [76]. Similarly, speed skaters exhibited increased jump height after eastward travel across 10 time zones [94]. Overall, the effects of long-haul travel on anaerobic power are inconsistent, depending on sport modality, travel direction, and individual characteristics. Temporary impairments, particularly in high-intensity efforts after eastward travel, are common, though some athletes may experience enhanced outputs in specific power tasks. Therefore, strength and conditioning coaches, exercise physiologists, and medical staff should monitor athletes’ anaerobic performance after travel and implement individualized recovery, training, and adaptation strategies to optimize post-travel performance.

#### 4.2.2. Strength

Strength responses vary by travel direction, sport modality, and circadian timing of assessments. Press–pull strength, at both slow and fast velocities, decreased after westward travel [25]. In elite athletes from multiple sports (e.g., track and field, swimming, cycling) traveling eastward, muscle strength declined for several days, exhibiting a circadian rhythm with the lowest values in the morning and peak strength in the early evening [35]. Among second-division American college basketball players, knee flexion strength decreased over a season of travel, accompanied by a reduction in vertical jump height [61]. In contrast, rugby players showed no change in muscle strength or range of motion after westward travel [46]. Conversely, in highly trained male rowers, eccentric velocity and jump height improved after westward travel [76], and speed skaters jumped higher after eastward travel across 10 time zones [94]. Therefore, sport scientists should monitor athletes’ muscle strength post-travel and implement individualized recovery and training strategies to optimize performance.

#### 4.2.3. Velocity

Maximal 30 m sprint and race times were unaffected in skeleton athletes and speed skaters after eastward travel [33,94]. Mean velocity during LCMJ increased after westward travel [76]. These findings align with a systematic review of elite athletes showing improved sprint performance after westward travel [77]. However, the same review reported negative effects on sprint performance in another group of athletes following westward travel [77]. Evidence on the effects of transmeridian travel on velocity is mixed: some studies report no change, while others show impairments or improvements, particularly after westward flights. These results suggest that jet lag effects on velocity depend on travel direction (typically worse after eastward travel) and sport type. Therefore, coaches and practitioners should closely monitor athletes’ speed-related performance after travel and apply individualized training and recovery strategies to maintain or optimize post-travel velocity.

#### 4.2.4. Aerobic Power and Capacity

Aerobic power and capacity are compromised by long-haul travel, and its recovery depends on individual differences, travel direction, and timing of subsequent training or competition. College swimmers traveling westward experienced a measurable decline in endurance performance [18], and similar reductions were observed in male professional soccer players after eastward travel across 11 time zones [50]. Sleep disturbances and accumulated travel fatigue further compromise aerobic performance, reducing maximal oxygen consumption ($\dot{V}$O_2max_). Aerobic power and capacity are particularly vulnerable to the combined stressors of time zone changes, sleep disruption, and travel-related fatigue. Therefore, exercise physiologists should monitor athletes individually and implement targeted interventions, including strategic training scheduling, sleep management, and recovery strategies, to minimize performance decrements in endurance sports.

#### 4.2.5. Coordination and Reaction Time

Coordination is impaired by travel [12], generally more after westward travel [29]. German Olympic gymnasts showed declines in coordination after westward travel across 6 time zones, while eastward travel had less effect [29]. Similar patterns were observed in athletes from multiple sports [12]. In fact, coordination and reaction time are particularly sensitive to long-haul travel. These disturbances are likely driven by circadian misalignment and fatigue, which can compromise fine motor control and responsiveness, critical for technical performance in sports requiring precision and rapid decision-making. Coaches should assess technical and cognitive performance, and implement training recovery protocols, timing strategies, and fatigue mitigation techniques to preserve technical performance.

#### 4.2.6. Sport Specific Tasks

Game performance in NBA, NFL, MLB, NHL, and rugby shows travel-related decrements. Westward travel often correlates with poorer performance, though eastward travel sometimes impairs outcomes. Most studies examining the effects of long-haul travel on physical performance used non-sport-specific tasks. However, several longitudinal studies in the USA and Australia assessed actual game performance and sport-specific statistics (e.g., free throws made, home runs, final match results). In the NBA, travel before a game negatively affected team performance, including win percentage, free throws, field goal percentage, and rebounds [13,53,69]. Travel direction was strongly correlated with performance, with westward travel linked to poorer outcomes [13,53,69], although some studies reported eastward travel associated with reduced performance [83,95]. In American football (NFL and NCAA), eastward travel impaired teams’ ability to win, while westward travel negatively affected overall performance [16,17,59]. One study found no effect of travel direction on match outcomes [53]. In the NHL, westward travel disadvantaged teams [53], but in general, travel negatively impacted overall performance regardless of direction [81]. In MLB, travel reduced team performance, with eastward travel having a greater negative effect than westward [52]. Outside North America, in rugby (NRL, Australia), travel adversely affected performance regardless of direction [84]. Overall, long-haul travel impairs sport-specific performance, with measurable effects on game statistics and team outcomes across professional leagues. Contrary to traditional assumptions that eastward travel produces more severe jet-lag symptoms, westward travel was frequently linked to poorer performance in game metrics. These decrements are likely driven by circadian disruption, fatigue, and travel-related stress, emphasizing the importance of strategic travel management in professional sports.

### 4.3. Integrated Pathways Linking Travel, Circadian Disruption, and Performance

Long-haul travel disrupts athlete performance through interacting, rather than isolated, mechanisms. Acute travel fatigue and jet lag often coexist and jointly disturb key physiological systems. These disturbances are closely linked to instability of the sleep–wake cycle and delayed recovery, which in turn constrain aerobic and anaerobic performance, strength, velocity, coordination, and sport-specific skills. At the same time, variability in performance outcomes across studies reflects differences in travel direction, number of time zones crossed, sport modality, and individual responses. Therefore, performance changes should be interpreted as the cumulative result of these interacting physiological disruptions rather than as isolated effects, reinforcing the need for targeted, individualized recovery and monitoring strategies after long-haul travel.

### 4.4. Limitations

Heterogeneous populations, small sample sizes, observational designs, and uncontrolled contextual factors limit generalizability. Direct comparisons across studies are challenging due to variations in sport, competitive level, travel direction, number of time zones, and performance measures. Additionally, inter-individual variability (e.g., chronotype, sex, and training status) may influence individual responses to travel and circadian disruption, which complicates the interpretation of group-level findings. Future research should incorporate stratified analyses, larger and more diverse samples, and standardized assessments of individual characteristics to better account for these moderators and improve the applicability of findings.

Of the studies included, 49% were rated as low risk of bias, 17% as moderate, and 34% as high, resulting in an overall moderate methodological quality. The predominance of observational, narrative, and case-report designs, together with moderate-to-high risk of bias, may inflate, obscure, or contribute to inconsistencies in reported effects on physiological and performance outcomes. The limited number of intervention-based studies constrains causal inference, so findings should be interpreted as indicative rather than definitive. These methodological considerations highlight the importance of applying the findings cautiously, tailoring monitoring and intervention strategies to each athlete’s context, as discussed in the following practical recommendations.

### 4.5. Practical Applications

**Figure 4:**
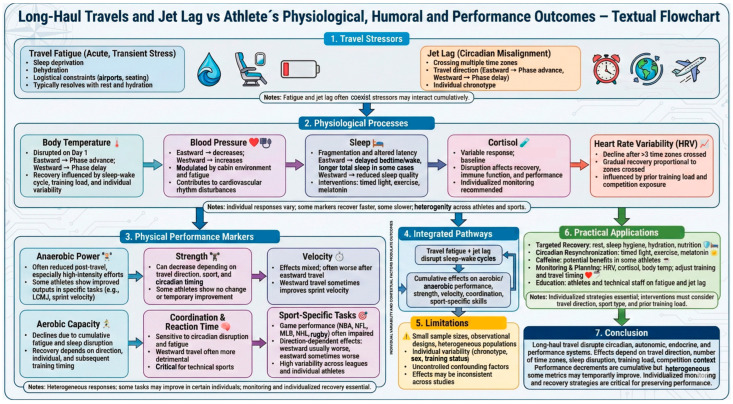
Textual Flowchart “Long-Travels and Jet Lag vs. Physiological, Humoral and Performance Outcomes”.

Long-haul travel and jet lag have multifactorial effects on physical performance, physiological markers, and athletes’ well-being. Coaches, medical staff, and sports managers should adopt preventive strategies and individualized monitoring to mitigate these effects. Trip planning should allow gradual adaptation to the new time zone. Physiological variables (e.g., HRV, cortisol, body temperature) should be monitored to evaluate recovery and readiness. Behavioral interventions, including sleep hygiene, strategic light exposure, proper nutrition, and hydration should be implemented, alongside education for athletes and technical staff to recognize early signs of travel fatigue and jet lag. Some physiological effects are well established, but other mechanisms, including hormonal imbalances, still need further confirmation in elite athletes. Likewise, although supplementation was not the experimental focus of most original articles analyzed, the recurring mention of caffeine in the included reviews underscores its potential relevance as a mitigation strategy; however, such interventions remain not fully conclusive. Figure 4 provides a schematic overview of the study’s main findings, illustrating the interactions between long-distance travel, jet lag, and the resulting physiological, humoral, and performance responses.

### 4.6. Future Research Directions

Despite the growing body of evidence on athletic travel, several gaps remain to be addressed to advance the field:

- **Female athlete representation:** There is an evident need for studies focusing on female athletes.
- **Standardization of performance metrics:** Standardized performance testing protocols are welcome;
- **Mechanistic links:** Future studies should move beyond descriptive observations to establish clearer mechanistic links between biological markers and sport-specific performance related outcomes;
- **Diversified interventions:** While light exposure and melatonin are well-studied, there is a need for randomized controlled trials (RCTs) exploring alternative interventions, such as nutritional ergonomics, blood flow restriction (BFR);
- **Between-sport and within-sport comparisons:** Comparative research is needed, for example, to determine if the social external timing cues (zeitgebers) and logistical demands of team sports result in different adaptation patterns compared to the highly individualized environments of individual sports.

## 5. Conclusions

Long-haul travel and jet lag appear to produce multidimensional effects on athletes by potentially perturbing circadian, autonomic, endocrine, and performance systems. The magnitude and trajectory of these disturbances are likely influenced by travel direction, number of time zones crossed, sleep disruption, prior training load, competitive context, and individual susceptibility. Although performance outcomes remain heterogeneous, evidence suggests that aerobic capacity, coordination, and technical execution may be particularly vulnerable, whereas anaerobic power and strength demonstrate variable responses, including temporary improvements in certain modalities and directions of travel. At the physiological level, disruptions in core body temperature, blood pressure, HRV, sleep architecture, and cortisol secretion often reflect misalignment between internal circadian rhythms and external environmental cues. These alterations may impair autonomic balance, recovery processes, and readiness to perform, potentially contributing to decrements in competition-specific performance and game statistics across multiple team sports, although null and mixed findings are also reported. HRV appears to be a sensitive non-invasive marker for tracking autonomic recovery, while endocrine responses, particularly cortisol, underscore the importance for individualized monitoring due to their variability. Overall, the available evidence suggests that travel-related performance changes are not solely determined by east–west directionality but likely reflect an interaction between circadian phase, competitive timing, home–away context, and logistical stress. Consequently, personalized management strategies, encompassing strategic scheduling, sleep and light manipulation, nutrition and hydration, training load adjustment, and athlete education, are likely beneficial to help mitigate the effects of jet lag, preserve performance, and optimize recovery. Future research should aim to refine mechanistic understanding, identify interindividual phenotypes of susceptibility, and develop evidence-based protocols tailored to sport modality, competitive calendar, and travel demands. However, given the heterogeneity in study designs, sports modalities, and athlete populations, these findings should be interpreted with caution, as they may not be directly generalizable to all sporting contexts.

## Figures and Tables

**Table 1:** Main databases and searches used to identify relevant articles.

	*n*
PUBMED (((((“jet lag”) OR (“travel fatigue”)) OR (“long travel”)) OR (“transmeridian travel”)) AND (Performance)) AND (athletes) Filters: Full Text	134
SCOPUS TITLE-ABS-KEY((((((“jet lag”) OR (“travel fatigue”)) OR (“long travel”)) OR (“transmeridian travel”)) AND (Performance)) AND (athletes))	11
WEB OF SCIENCE ALL = ((((((“jet lag”) OR (“travel fatigue”)) OR (“long travel”)) OR (“transmeridian travel”)) AND (Performance)) AND (athletes))	139

**Table 2:** Study characteristics.

Reference	Study Design	Participants	Sport Modalities	Travel Details	Supplementation	Main Results	Conclusions
Winget et al. (1985) [15]	Review	n/a	n/a	n/a	n/a	n/a	Athletic performance undergoes significantly daily oscillations under the control of the circadian system. The window for optimal performance is between 12:00 and 21:00.
Loat et al. (1989) [3]	Review	n/a	n/a	n/a	n/a	n/a	Circadian rhythm disruptions negatively affect performance and require consideration in competition
Hill et al. (1993) [25]	Review	USA Women’s National Soccer team (*n* = 7); Healthy individuals (*n* = 19)	Soccer	WC NA to Taiwan; NA to West Europe; Europe to NA	n/a	Mood disturbances (↑ fatigue/confusion; ↓ vigor) in first 1–2 days; grip strength ↓; anaerobic power and work capacity ↓; mainly Days 1–2 post-travel	Rapid travel across 6–8 time zones temporarily disrupts mood, sleep and anaerobic performance with recovery occurring within a few days
Jehue et al. (1993) [17]	Observational design	All 28 NFL Teams	American Football	All in America (west to east; east to west)	n/a	Day games: home 56.6%, away 43.8%; night games: home 61.9%, away −23.8% change. Away teams lost more in East/Central; WC teams kept high home win %	Home field advantage is strong, but West-to-East travel impairs performance, whereas East/Central teams are less affected. Adaptation appears faster after westbound travel
Eichner (1994) [18]	Review	college swimmers (22 men, 18 women); 27 National Football League teams	Swimming; American Football	Wisconsin to Hawaii and back (4 time zones)	n/a	Travel across 4 time zones had minimal impact on mood, soreness or perceived effort in swimming. WC teams lose more in day games when traveling east but win night games regardless location	Circadian rhythms significantly influence health and performance and should guide training and daily activity timing
Smith et al. (1997) [16]	Observational design	NFL teams from 1970 to 1994 seasons	American Football	n/a	n/a	WC teams won more overall (63.5%) and on MNF (59–71%). EC MNF home wins (43.8%) but increases vs. non-WC opponents (67.5%)	Circadian timing can affect performance as much as home/away status; competing near peak time may be more beneficial than full time zone acclimation
Reilly et al. (1997) [4]	Review	Swimmers; Rugby players; Military personnel; British; Olympic squad members; American footballers	Swimming; Rugby; American Football	England to Australia (eastward, 6 time zones); London to Tallahassee (westward, 5 time zones)	Melatonin Hypnotics	Muscle strength affected more by time-of-day than short sleep; performance drops lasted ~5 days. Simpler tasks adapted within 3 days; eastward travel caused greater performance decline than westward	Fatigue from travel is worsened by jet lag and environmental stress; athletes benefit from behavioral, not pharmacological strategies
Manfredini et al. (2000) [26]	Quasi-Experimental design	elite athletes of the Italian National Biathlon Team (*n* = 8 men, mean age 25.9 years; *n* = 4 women, mean age 22.5 years)	Biathlon	Milan to Japan (eastward, 8 time zones)	Melatonin	Men with delayed body temperature peaks showed little change after flight; melatonin advanced peaks afternoon. Women showed an 8 h shift after flight; melatonin had no effect. No adverse effects or sleep disturbances were reported	Standardized melatonin intake may variably affect biological rhythms, with incomplete resynchronization influenced by inter-individual variability
Straub et al. (2001) [27]	Experimental	(*n* = 15) were male (*n* = 12) and female (*n* = 3) Finnish junior elite track and field athletes	track and field	Helsinki to Marietta (westward, 7 time zones)	n/a	No significant effects of treatment or group were observed on TMD, HR or jet lag ratings. Total jet lag scores: westward 160, eastward 126.5	No significant effects observed; emphasizing sleep is important, particularly alongside jet lag
Cardinali et al. (2002) [28]	Case report	Male professional soccer players and their coaches from Boca Juniors Club (*n* = 22)	soccer	Buenos Aires—Tokyo (westward, 12 time zones)	Melatonin	Sleep largely unchanged; awakenings ↑ (days 1 and 3), sleep latency ↓ (days 2,6,8). Mean resynchronization 2.13 ± 0.88 days vs. expected 6 days (significant)	Melatonin plus timed light and exercise accelerates resynchronization; individualized dosing improves jet lag outcomes
Waterhouse et al. (2002) [2]	Observational Study	Athletes (*n* = 51) Trainers (*n* = 18) Academics (*n* = 16)	n/a	UK to Australia (eastward, 10 time zones)	n/a	Jet lag symptoms decreased over days in Australia. Older subjects experienced less fatigue, men slept later and had less early fatigue. Fitter/repeat travelers reported worse jet lag. Later arrivals experienced less jet lag and better appetite. Phase-advance ↑ jet lag on final days	Flexibility in sleep, time of arrival, age, and adjustment direction affect jet lag and fatigue; phase-advance adjustments may worsen symptoms, especially early in the day
Lemmer et al. (2002) [29]	Observational Study	Members of the German Olympic gymnastics team (*n* = 15)	Athletics	Frankfurt to Atlanta (westward, 6 time zones) Munich to Osaka (eastward, 7 time zones)	n/a	Jet lag peaked first 3 days; eastward flights worse initially; training less affected than westward. West: BP, HR, cortisol, melatonin, temperature, GS, disrupted up to 11 days. East: HR stable; cortisol, melatonin, temperature, GS disrupted; melatonin peak delayed ~4 h	Time zone changes disrupts cardiovascular rhythms and performance; ≥2 weeks needed post westward flight; individual differences must be considered
Waterhouse et al. (2004) [8]	Review	n/a	n/a	n/a	n/a	Travel stressors impair health and cognition; frequent travelers at higher risk. Stress management, sleep hygiene, hydration, nutrition and activity reduce effects	Jet lag impairs mood, training and performance; preparation, sleep, melatonin and light exposure are key countermeasures, especially in endurance sports
Reilly et al. (2005) [10]	Review	Elite athletes, sedentary controls, and military personnel and airline crew members	Soccer; rugby; swimming; cycling; tennis; badminton; golf; American football	UK to Florida (westward,5 time zones); UK to Australia (eastward, 10 time zones)	Melatonin; caffeine; hypnotic agents and other banned stimulants (amphetamines, modafinil)	Jet lag disrupts sleep, cognition and performance (worse eastward, 5–7 d). Circadian misalignment impairs strength/power; light and sleep shifts help; melatonin inconsistent	Jet lag hinders performance; behavioral strategies > drugs; athlete/staff education is essential
Postolache et al. (2005) [30]	Review	American footballers; Olympic athletes; Sedentary subjects; Swimmers; Female soccer players; Rugby League players	American Football; Swimming; Soccer; Rugby	n/a	stimulants (amphetamines, methylphenidate, ephedrine, cocaine); caffeine; pseudoephedrine; melatonin	Adjustment matched zones crossed; eastward worse. Pre-shift training helped. Symptoms: appetite loss, constipation. Women distinct, staff at higher CV risk. Body temperature readapted before BP	Jet lag is transient, training continues, but with reduced quality of performance. Behavioral strategies + rehydration is effective; drugs are limited. Educate athletes and staff to optimize adaptation
Armstrong (2006) [31]	Review	n/a	Soccer	Transmeridian travel	n/a	Performance peaks: 14–18 h (physical), 12–15 h (mental). Jet lag often causes sleep loss, slight thermoregulation impairment. Food timing affects sleep; pre-travel training shifts help. Melatonin ± caffeine ↓ symptoms	When athletes experience jet lag, caffeine consumption and adjustments of meal size/composition may be helpful
Milne et al. (2007) [32]	Case report	Rugby Player (*n* = 1)	Rugby	Great Britain to New Zealand (eastward, 12 time zones); Great Britain to New Zealand (westward, 12 time zones)	Melatonin; Triazolam	Performance in the final was up to its usual high standard, directing play effectively, plus he kicked a conversion and three penalty goals	Appropriate advice and selective use of medications, travel across many time zones do not need to be associated with a detriment in performance in the sporting arena
Bullock et al. (2007) [33]	Observational Study	Elite Australian skeleton athletes (*n* = 5) Elite Canadian skeleton athletes (*n* = 7)	Skeleton	Canberra, Australia to Calgary, Canada (eastward, 4 time zones)	n/a	Cortisol ↓ 67% day 1, 47% day 2 (NS). Sprint and urine SG unchanged. Jet lag ↑ days 1–4, 7; improved on day 11. Hunger, motivation, concentration, tiredness remained stable	Eastward travel: cortisol rhythm disrupted, jet lag ↑ 7 days; sprint performance unaffected. Circadian resynchronization ~1 day/time zones (varies individually)
Reilly et al. (2007) [34]	Narrative review	n/a	n/a	n/a	Melatonin; caffeine; probiotics	Jet lag ↓ sleep, mood, digestion, performance. Nutrition strategies ↓ symptoms, ↑ hydration levels. Travel risks: limited food, hygiene, culture, buffet temptations, diarrhea (up to 60% of travelers)	Plan and educate athletes. Key: meals, hydration, light, hygiene. Behavioral strategies are the macronutrients. Drugs/supplements ⟶ cautions. Adaptation leads to optimal performance
Reilly et al. (2007) [11]	Position statement	n/a	n/a	n/a	n/a	n/a	Education ⟶ informed travel choices. Behavioral adjustments preferred over drugs for time-zone adaptation
Meijer et al. (2008) [35]	Narrative review	Elite athletes; Healthy volunteers; General population	Track and field; Swimming; Cycling; Soccer; Tennis; Badminton; High Jump/Long Jump	eastward to Beijing (≥6 time zones)	Melatonin	Performance peaks early evening, lowest morning. Eastward jet lag ↓ performance; disrupts BP, body temperature, strength rhythms. Sleep loss ⟶ impaired hormonal balance (↑cortisol, ↓ GH), glucose metabolism and tissue recovery	Circadian misalignment ↓ performance; adjust clocks ≥ 2 weeks pre-event. Light, sleep, local meals, no alcohol/caffeine ⟶ ↓ jet lag and competitive readiness ↑
Dranitsin (2008) [36]	Experimental	(12 males and 1 female); age 17.7 years, s = 0.8; height 1.89 m, s = 0.06; body mass 88.9 kg, s= 13.1; training history 4.0 years, s = 0.9	Rowing	Kiev, Ukraine to Beijing, China (eastward, 5 time zones)	n/a	Beijing ↑ temperature/humidity vs. Kiev. HRV stable days 1–3, ↓ standing position days 4–5, partial recovery day6, ↓ again days 8–10, baseline day 13. Competition ⟶ no HRV change. Standing HRV was strongly related to humidity. Supine HRV unaffected	Standing HRV adapts first 5 days post 5 time zone shift; supine HRV correlates with prior training load
Montaruli et al. (2009) [37]	Case study	Amateur level marathon runners (*n* = 18 males)	Athletics	Milan to New York (westward, 5 time zones)	n/a	Sleep parameters and efficiency ⟶ no group differences post-flight. Cosine peak: day 1 differences persist; day 2 ⟶ CG/ETG 20–30 min delay, MTG 2.5 h delay. Questionnaire ⟶ no worsening; CG vs. marathon groups NS	Evening physical activity ↑ sleep quality; shifts circadian rhythms ⟶ faster westward adaptation. Scheduled exercise ↓ jet lag sleep disturbances
Pipe (2011) [7]	Review	n/a	n/a	n/a	n/a	n/a	Travel planning + sleep/light/activity ⟶ restore sleep–wake cycles. Training timing/intensity ↓ injury, ↑ circadian reset
Chapman et al. (2012) [38]	pre–post intervention comparison with a control group	national team skeleton athletes (World Cup athletes and America’s Cup athletes) (*n* = 12)	Skeleton	Canberra, Australia to Canada (eastward, 4 time zones)	n/a	Contact time unchanged. Flight time ↓ 6% day 9; BDJ ↓ 4.2% day 5. CMJ height ↓ 1–2 days; CMJ velocity/power stable. SJ velocity/power ↓ post-flight; EUR power ↑ days 2,4,7	Long-haul travel ↓ acyclic power; velocity/power EUR disrupted 48 h. Slow SSC more affected; BDJ minor changes. Arrive ≥ 5 days pre-competition
Schobersberger et al. (2012) [5]	Report	n/a	n/a	n/a	Melatonin	Travel related thrombosis is rare but should be considered. Crossing 1–2 time zones ⟶ minimal jet lag. Eastward ⟶ harder sleep. Adjustment: west 1 day/zone, east 1–5 days/zone	Prioritize sleep and recovery before flight. East ⟶ sleep/wake 1 h earlier; West ⟶ 1 h later. Hydrate, avoid alcohol/caffeine, light exercise. Align sleep and meals to local time. Pretreatment or melatonin if ≥5–8 time zones
Samuels (2012) [6]	Review	n/a	n/a	n/a	Melatonin	n/a	Jet lag and travel fatigue ⟶ major disturbances. Structured travel program + monitoring ⟶ symptoms ↓, performance ↑
Lee et al. (2012) [14]	Descriptive review	n/a	n/a	n/a	n/a	Behavioral > pharmacological strategies. Sleep, light timing, ± melatonin ⟶ adaptation	Travel ⟶ physical and cognitive function ↓; mood, cognition, performance are impaired by jet lag
Forbes-Robertson et al. (2012) [12]	Review	Elite and sub-elite athletes from various sports, plus data from general population and shift workers	Soccer; rugby; swimming; cycling; tennis; American football; endurance skill-based sports	Eastward and westward intercontinental travel crossing multiple time zones	Caffeine; melatonin; prescribed hypnotics (zopiclone, temazepam)	Circadian misalignment ↓ performance, reaction skill, cognition. East travel slower. Light ⟶ strongest cue; melatonin aids sleep; caffeine ↑ alertness, may ↓ recovery. Macronutrient timing has limited effect	Circadian disruption ↓ performance/recovery. Behavioral strategies primary; drugs selective. Education + individualized plans are essential to performance
Leatherwood et al. (2013) [39]	Review	n/a	n/a	n/a	n/a	n/a	Air travel effects are hard to study; small samples, variable measures, limited generalizability
Thompson et al. (2013) [40]	parallel group randomized controlled trial	elite female soccer players from a national team (*n* = 22; 26 ± 4 years; 65.1 ± 5.9 kg; height of 1.71 ± 0.05 m)	Soccer	East coast of USA to Lisbon, Portugal (westward, 5 time zones)	Bright Light	LG: jet lag ↑ first 24 h ⟶ over 4 days ↓; function worst 8–12 h, best improvement on day 2. Sleep night 1 was best. Temperature ↑ day 1. GS differed, unclear pattern	Light ↑ body temperature acutely; no jet lag relief in elite female footballers (west 5–8 time zones). Effects mainly acute, not chronobiological
Stellingwerff et al. (2014) [41]	Review	aquatic athletes	Swimming	n/a	n/a	Melatonin ↓ sleep onset ~7 min; well tolerated; unclear impact on performance/recovery	Adequate hydration, carbs, iron, fluid management, hygiene ⟶ mitigate environmental stress during travel
Fuller et al. (2015) [42]	cohort study	All players from nine core teams competing in the Sevens World Series from 2008/2009 to 2013/2014	Rugby	n/a	n/a	Injury incidence is similar across travel categories; Category C ↓ injuries. No differences in type, cause, timing or activity. One country showed potential performance effect	4 days recovery are generally sufficient to recover; long-distance travel ⟶ no ↑ in overall, muscle or forwards’ injury risk
Fowler et al. (2015) [43]	Observational Study	Professional Australian football (soccer) players (*n* = 16)	Soccer	Australia to Japan (northbound, 1 time zone)	n/a	Away travel ↓ training load/intensity/speed; ↓ sleep and wellness; ↑ jet lag; ↑ fatigue and soreness; stress/mood were unaffected. High jet lag in fewer first-team appearances	Minimal time-zone travel ⟶ little effect; sleep disruption and competition fatigue ⟶ larger impact; experience athletes ↓ impact; avoid early/late travel
Simmons et al. (2015) [44]	Review	n/a	n/a	n/a	n/a	n/a	Short trips ⟶ naps/caffeine/limited use of sedatives; long trips ⟶ rapid sleep–wake synchronization, timed melatonin/light; medications may be helpful but carry side effects
Silva et al. (2016) [45]	cross-sectional survey	male kite surfers (34.3 ± 8.8 years) *n* = 94	kite surf	n/a	n/a	SM ⟶ farther travel, earlier arrival, ↑ fluids/fruit, broader jet-lag strategies; NoSM ⟶ mainly eating/drinking. No meds. More training/sleep/water ⟶ fewer travel effects	Travel effects ⟶ distance, sleep, hydration, nutrition education needed. Mitigate jet lag: early arrival, ↓ training, sleep hygiene. Eastward travel ⟶ worse symptoms, ↓ performance
Fowler et al. (2016) [46]	Observational Study	male professional rugby league players (*n* = 18)	Rugby	Sydney, Australia, to London, UK (westward, 11 time zones)	Melatonin	Jet lag↑ post-2, 6, 8; sleep/fatigue improved post-8; UR symptoms ↑ post-6; wellness stable; strength/soreness unchanged; training load ↓ post-travel	Strength and ROM recover in 24 h; training preserves wellness; jet lag, sleep, UR symptoms ↑ ≤8 days, use sleep hygiene for recovery
Fullagar et al. (2016) [47]	Observational study	Elite male football players (*n* = 15)	Soccer	London, United Kingdom to Montevideo, Uruguay (westward, 4 time zones)	n/a	NS changes from baseline in perceptual measures; jet lag ES large on day 2, moderate on day; sleep restfulness ES moderate on day 6	LH travel ↓ sleep, minimal recovery impact; rebound ↑ sleep on first night
Fowler et al. (2017) [48]	Case study	male professional football players from the Australian national football (*n*= 22; Mean ± SD; age 26 ± 4 y, height 180 ± 6 cm, body mass 75.8 ± 6.5 kg)	Soccer	Sydney, Australia to Vitoria, Brazil (eastward, 11 time zones)	Melatonin	Jet lag ↑ post 1–4; sleep ↓ during travel/arrival; wellness ↓ post-travel week	Eastward travel ⟶ sleep disruption, jet lag ↓ wellness and readiness
Williams et al. (2017) [49]	Review	n/a	n/a	n/a	n/a	n/a	Sleep, light, nutrition, exercise, hygiene and travel planning mitigate travel effects
Fowler et al. (2017) [50]	Observational Study	healthy, physically trained men (*n* = 10)	n/a	Sydney, Australia to Doha, Qatar (westward, 8 time zones)	n/a	Eastward travel impaired peak force, sprint speed, YYIR1 performance, sleep, motivation and hydration more than westward; most effects recovered by day 4	Lower-body power recovers within ~96 h after long-haul travel, but eastward flights disrupt sleep, jet lag, fatigue and sprit performance more than westward, especially in the first 48–72 h
Kölling et al. (2017) [51]	Observational Study	Rowers (male *n* = 30, 190.6 ± 7.5 cm, 86.3 ± 10.9 kg; female *n* = 25, 176.8 ± 6.3 cm, 71.3 ± 6.3 kg; mean age 17.8 ± 0.5 years)	Rowing	Germany to Brazil (westward, 5 time zones)	n/a	Fatigue peaked on the first evening post-travel; general and sport-specific recovery improved in Brazil, with most jet-lag symptoms decreasing over subsequent evenings	Jet lag peaked on arrival and persisted through day 6; early sleep times and reduced daylight delayed adaptation. Recovery ↑ by day 6
Song et al. (2017) [52]	Observational Study	Major League Baseball teams	Baseball	n/a	n/a	Winning%: Home teams +3.9%; eastward travel worse than westward; Offense: eastward travel ↓ doubles, triples, stolen bases; ↑ double plays; westward ↓ stolen base attempts; Defense: eastward travel ↓ performance; westward ↑ triples allowed; Away-team slugging: NS effects of travel direction	Jet-lag effects on winning percentage and runs scored were generally stronger after eastward travel.
Roy et al. (2018) [53]	Observational Study	Athletes of al teams competing in NBA, NHL and NFL	Basketball; American football; Ice Hockey	n/a	n/a	NBA: Westward evening ↓ win%, eastward evening ↑ win%; time zones explain 18.5%; no afternoon effect. NHL: Westward ↓ win%, eastward no advantage; time zones explain 23.2%; no afternoon effect. NFL: No travel direction effect; time zones explain 11.3%; no afternoon effect	Evening away games show a significant link between winning % and number of time zones traveled in NBA, NHL and NFL. Westward travel in the evening is disadvantageous, with greater effects as time zones increase. Teams performing closer to their circadian peak have an advantage, highlighting the role of circadian rhythms in sports performance
Stevens et al. (2018) [54]	Prospective cohort study	Masters level triathletes (age: 48 ± 14 years, height: 172 ± 11 cm, body mass: 72 ± 11 kg) (*n* = 12)	Triathlon	Australia to Hawaii (eastward, 21 time zones)	n/a	Sleep duration: ↓ during flight, ↑ Night 1, returned to baseline, ↓ night before competition; sleep quality and efficiency unchanged; 25% of athletes developed mild to severe illness 3–5 days post-arrival; sIgA and cortisol unchanged from day 2 onward	LH NE travel ↑ sleep disruption and fatigue, but immunity and stress markers remain stable; recovery occurs within 48 h
Thornton et al. (2018) [55]	Longitudinal research	National wheelchair basketball athletes (*n* = 11)	Wheelchair basketball	USA (eastward travel) and Australia (westward travel) crossed up to 7 time zones to Manchester, UK; Europe crossed at least one but no more than 2 time zones to Manchester, UK	n/a	Long trips ↑ early bedtime/get-up and naps; ↓ vigor, ↑ jet lag/fatigue; Short trips ↑ vigor post 1–5	LH trips ↑ subjective jet lag, ↓ vigor and ↑ fatigue vs. short trips; subjective effects exceed objective sleep changes; adequate recovery time is needed before competition; circadian adjustment rates for Paralympic athletes may require re-evaluation
Silva et al. (2019) [56]	Narrative review of the literature	International team soccer players	Soccer	n/a	n/a	n/a	Jet lag arises after >3 time zones crossing; resynchronization depends on direction, cues and individual factors. Usually benign but may harm health and performance
Halson et al. (2019) [57]	Review	n/a	n/a	n/a	Melatonin; caffeine	Melatonin ↓ jet lag but had side effects; caffeine ↓ fatigue, mixed sleep effects; Argonne diet ↓ symptoms (1 study)	Pre-sleep diet (CHO, caffeine, alcohol, fluids) influence sleep; probiotics ↓ illness; antibiotic use is unclear; nutrition issues = scarcity or overeating
Broatch et al. (2019) [58]	Quasi-experimental	elite Australian female volleyball athletes (mean 6 SD: age, 25 6 2 years and range 22–27 years; body mass, 78.9 6 4.5 kg; and years competing at the international level, 5 6 2 years) *n* = 12	Volleyball	Canberra, Australia to Manila, Philippines (westward,2 time zones)	n/a	COMP vs. CG ⟶ CMJ ↑ (24–48 h), ↓ calf girth, altered SBP, HR, SO_2_; jet lag ↑ (+12 h); no major effects on mood, fatigue, soreness or coagulation markers	Compression socks ⟶ ↑ performance, wellness ↑, ↓ swelling, ↓ CV strain, stabilize SO_2_; no coagulation issues
Fullagar et al. (2019) [59]	Observational Study	American Football College Teams (NCAA)	American Football	n/a	n/a	Home ⟶ +5.3 points advantage; Penalty yards ⟶ no clear differences; Away < 483 kms ⟶ minimal effect; Away > 484 kms ⟶ moderate disadvantage; Crossing ≥ 1 time zone ⟶ −5 points disadvantage; East travel ⟶ no clear disadvantage; West travel ⟶ −7.5 points disadvantage	Playing at home in NCAA football is worth +5 points. Away travel > 483 km imposes a—point disadvantage, further exacerbated (−7.5 points) by westward time-zone travel. No clear disadvantage with short travel (<483 km) or eastward travel.
Lo et al. (2019) [60]	Observational Study	Super Rugby Teams from 2006 to 2017 season	Super Rugby	n/a	n/a	Travel effects trivial-moderate; East ↓ overall; West mixed (↑ tries, ↓ carries); small KPI differences after East	East LH travel ↓ performance/KPIs; Westward was unchanged or slight ↑. LH travel ↓ players and team KPIs; Eastward more detrimental than Westward
Atalag et al. (2019) [61]	Quasi-experimental	Division II university men basketball players (MBB) (age = 21.32 y ± 1.7 y; body weight 98.99 kg ± 16.15, total body fat = 16.81% (*n* = 36); age-matched men full-time university students (CT) (age = 22.67 y ± 1.2 y; body weight = 79.51 kg ± 17.1, total body fat = 20.37% ± 6.1 (*n* = 37)	Basketball	Hawaii to the mainland United States (eastward, 2 to 5 time zones)	n/a	MBB ↑ fat, waist/WHR, ↓ knee flexion, ↓ vertical jump, ↑ RHR and BP, ↑ cortisol; CG NS changes in body composition, performance, CV markers; ↑ vertical jump; BMD unchanged in both groups	East travel ↑ cortisol and trunk fat; ↓ knee strength in student athletes due to circadian disruption, cabin environment, limited movement and combined travel, academic and competitive stressors
Lo et al. (2019) [62]	Observational Study	Super Rugby Teams from 1996 to 2017 season	Super Rugby	n/a	n/a	Over 21 years: Wins ↑ east > west; away disadvantage ↓; points ↑; travel effect trivial-positive (east), unclear (west)	Continuous LH travel ↓ individual/team performance; over time, teams improved travel management, ↓ travel effects
Lastella et al. (2019) [63]	Case study	male professional soccer players (mean ± SD: age 25.2 ± 3.2 years, height 182.8 ± 5.2 cm, body mass 84.6 ± 7.4 kg; *n* = 7)	Soccer	Adelaide, Australia to Hiroshima, Japan (eastward, 1.5 time zones)	n/a	Location type affected bedtime, time in bed and total sleep time. Bedtime was later during flights vs. Adelaide and Hiroshima. No differences in get-up time, sleep latency, sleep efficiency, fatigue, movement, or subjective sleep quality	Flights disrupted sleep time and duration in professional soccer players. Sleep onset was later (+3.5 h), total sleep reduced (−3.h), quality poorer vs. home/away. Likely due to congested schedules and multiple flights, not minor time zone changes
Roach et al. (2019) [64]	Experimental	International-level athletes *n* = 11 (men from a wrestling team *n* = 6; mean age 5 20.2 6 0.8 years [age range: 19–21 years]; height5 176.1 6 7.6 cm; mass 86.3 6 17.9 kg, and women from the national fencing team *n* = 5; mean age5 23.6 6 2.7 years [age range: 21–28 years]; height5 169.4 6 4.0 cm; mass 59.1 6 5.4 kg)	Wrestling	Tokyo, Japan to Rio de Janeiro, Brazil (westward, 12 time zones)	8 mg of ramelton	Rio: TIB ↑, Experimental group sleep efficiency ↑ 1st to 3rd nights; SOL ↓; morning tiredness ↑ 1st night	Light + sleep + ramelteon ⟶ better sleep, less strain post LH travel
van Rensburg et al. (2020) [65]	Systematic review	Athletes	n/a	n/a	n/a	Little high-quality evidence exists for interventions managing travel fatigue or jet lag; exercise can induce circadian phase shifts, but timing is critical; sleep hygiene is essential; nutrition may help reducing jet lag symptoms; evidence for exogenous melatonin is weak; benzodiazepines may improve sleep quality and accelerate circadian readjustments (in some cases)	Caffeine ↑ alertness but delays circadian rhythms; Tasimelteon dose-dependent sleep/alertness effects, memory ↓; glucocorticoids realign circadian rhythms; antihistamines not advised; evidence in athletes is limited
Zubac et al. (2020) [66]	Narrative Review	Athletes (e.g., kite surfers, endurance athletes, rugby players, gymnasts); Healthy volunteers; Military aviators	Marathon; triathlon; volleyball; skeleton; rugby; gymnastics; combat sports; sailing	Europe to Middle East; Australia to Canada/UK/USA; crossing multiple time zones (up to 12 h difference)	Electrolyte; caffeine	Flight ↑ water loss; fluid intake often ↓; plasma volume sometimes ↓; impact on performance is unclear	LH travel ⟶ probable dehydration; combined effects with jet lag are unclear; hydration advice largely anecdotal
Nikolaidi et al. (2021) [67]	Commentary	Athletes	Athletics	n/a	n/a	BFR ↑ mTOR, corticomotor excitability, cortical activation ⟶ faster reactions	BFR is safe; can ↓ jet lag via physiological adaptations
Augusto et al. (2021) [68]	Observational design	male players from one club participating in the 1st Division of the Brazilian soccer championship (*n* = 20; aged, 27 ± 5 years; height, 180.5 ± 6.9 cm; body mass, 74.8 ± 7.8 kg)	Soccer	All in Brazil (short travel games with travel < 520 km; long-travel games with travel > 520 km)	n/a	TD ↑ in losses; sprint ↑ in draws; coach change ↓ running, HI sprint distance, HI actions, deaccelerations; long travel ↓ HI sprint distance; short travel NS	Long trips ↓ recovery and intense actions; travel factors also affect performance; running performance ↓ with long travel
Charest et al. (2021) [69]	Observational design	NBA Players from season 2013 to 2020	Basketball	n/a	n/a	Home-Away = Away-Away; win%: Away-Home 54.4%, Away-Away 39.2%, Home-Away 36.8%; travel ↓ impact except Away-Home; sequences ending home: travel ↓ results; eastward travel ↑ wins vs. west/no time zone change	Time zone travel ↓ recovery/mindset; home return ↑ wins (15–18%); distance effects vary by sequence; Away-Home ↑ sleep/circadian risk; long flights + inactivity ↓ O_2_ ⟶ wins ↓; eastward travel ↑ win% vs. west; fatigue/circadian disruption depend on distance
Fowler et al. (2021) [70]	randomized, matched-pairs design	healthy, physically trained males (*n* = 20)	n/a	Doha, Qatar to Sydney, Australia (eastward, 8 time zones)	n/a	INT ↑ CMJ peak/height, ↑ 5/20 m sprint (at 17:00 h); T-test/Yo-Yo NS; travel/post travel ↑ sleep duration/efficiency, earlier sleep; motivation/mood ↑; CG worse day 3; jet lag NS	Light + sleep hygiene ↓ sleep disruption, ↑ mood/motivation, ↑ CMJ; baseline recovery 72 h (INT) vs. 96 h (CG); sleep timing ↑ duration (2.5 h); jet lag NS
Janse van Rensburg et al. (2021) [71]	Review	Professional and elite athletes	Basketball; football; rugby; track and field	Short (<3 h) and long (>3 h) travel; trans meridian (east–west or west–east) and trans latitudinal (north–south)	Melatonin	Travel fatigue: no high-quality evidence; strategies ⟶ sleep, planning, hydration/nutrition, illness, prevention; Jet lag: limited evidence; strategies ⟶ light, melatonin, sleep/exercise adjustment, nutrition	Travel fatigue/jet lag ↓ performance/health; expert consensus guides management; future studies ⟶ validated tools, RCTs, individualized strategies, physiological markers (melatonin, CBTmin)
Lalor et al. (2021) [72]	Observational Study	Elite female cricketers from the national team (*n* = 11)	Cricket	Melbourne, Australia to Chennai, India (westward, 5.5 time zones)	n/a	AM jet lag ↑ days 1–3; PM jet lag ↑ day 2; in-flight ↑ sleep efficiency ⟶ ↓ fatigue; stress ↑ total average wake; soreness ⟶ ↑ wake and ↓ efficiency; well-being ⟶ longer wake bouts; higher AM jet lag ratings ⟶ ↓ sleep efficiency and ↑ total wake	In-flight sleep ↑; during competition ⟶ ↓ sleep; planning departure ⟶ better recovery, ↓ jet lag; stress/soreness ↓ efficiency, ↑ wake; sleep fragmentation possible despite low stress; avoid early AM activity to optimize sleep
Leduc et al. (2021) [73]	Observational Study	male international rugby sevens players from an international team based in Europe (*n* = 17)	Rugby	eastward and westward	n/a	Pre-tour ⟶ TST ↓; WASO ↓ pre-tour; sleep quality ↓ tourn. 1–2 + relocation; eastward ⟶ earlier sleep onset; westward ⟶ earlier wake; tourn.1 westward ⟶ sleep ↓; eastward pre-competition ⟶ quality/efficiency ↑; westward pre-competition ⟶ WASO ↑	LH travel did not negatively affect short-term sleep; tourn/relocation ⟶ biggest sleep ↓; pre comp > pre-tourn sleep; east vs. west ⟶ sleep differences; direction unclear, westward ↓ quality
Lo et al. (2021) [74]	Qualitative description	(S&C) Coaches (*n* = 3) or Medical Doctors (*n* = 5) from Super Rugby Teams from 2016 season (*n* = 8)	Super Rugby	n/a	n/a	Main issues: fatigue + sleep disruption; all teams use strategies ⟶ same choices, different implementation; some rely on sleep meds; naps variably allowed; flight planning common; practice > literature; one clinical/individualized vs. one humanistic	Travel ↓ performance; teams use mixed strategies (experience > literature); gap science-practice; travel management = space for innovation ⟶ ↑ performance and cohesion
Smithies et al. (2021) [75]	Longitudinal Observational Study	male professional rugby players (*n* = 37)	Super Rugby	Westward	n/a	6% ↑ sleepiness, 39% ↓ sleep quality; chronotype: 50% intermediate, 43% morning, 7% evening; Day 7: TASO ↑ 143 min, TST ↓, TAW ↑ (day 4–5)	LDTT ⟶ sleep timing shifts, TST stable; avoid AM activities/flights post-match; westward AM flights ↓ adaptations; TST ↓ short term, normalizes in 2 d
Everett et al. (2022) [76]	Prospective single-group observational pre-post measures	Highly trained male rowers (*n* = 21, 23.7 ± 1.4 years, 190.9 ± 7.5 cm, 86.9 ± 9.9 kg)	Rowing	Canberra, Australia to Milan, Italy (westward, 9 time zones)	n/a	Westbound travel ⟶ JH ↑10.3% (NS), Mean Velocity ↑ 4.6%, EMV ↑, Dip ↑ 7.4%, JH:Dip ↑5.9%, Power ↑5.5%	Westbound travel ⟶ concentric/eccentric velocity ↑, mean power ↑, eccentric displacement ↑; CMJ ↓
Rossiter et al. (2022) [77]	Systematic review	elite athletes	n/a	n/a	n/a	14 studies (197 athletes); NOS 6 ± 1. Jet lag ↑ post LH, less severe westward. Temperature, BP, cortisol disturbed ≤11 days, sometimes recover at day 3. Jump and sprint performance: ↓ skeleton, ↑ rowers	Perceived jet lag ↑, sleep/psychometrics minimally affected; recovery ⟶ 1 day/time zone; performance effects unclear: GS ↓, complex tasks =); symptom/recovery variability ⟶ sex, age, chronotype; mechanisms poorly understood
Flatt et al. (2022) [78]	Observational study	Great Britain athletes selected for Olympic Summer Games (*n* = 12 men; height = 185 [7] cm; weight = 91 [7] kg; maximum aerobic speed = 4.60 [0.15] m·s^−1^)	Rugby	Grain Britain to Brazil (westward, 4 time zones)	Caffeine supplementation (100– 200 mg)	LnRMSSD average ↓ days 4–7; LnRMSSD ↑ day; sleep/soreness/recovery/energy significant ↓, mood NS; recovery ↓ day 1, ↑ day 13; energy ↑ day 13	Tournament/travel ⟶ HRV ↓, returns ↑; AM activity westward ⟶ circadian resynchronization; low-intensity > high-intensity for HRV recovery; CWI ⟶ parasympathetic ↑; elite athletes: HRV ↑ and stable; less experienced players = more vulnerable
Lever et al. (2022) [79]	Case study	junior national level netball players (*n* = 11)	netball	Johannesburg to Sydney (eastward, 11 time zones); Sydney to Johannesburg (westward, 11 time zones)	n/a	TIB ↑ on match and pre-travel vs. travel and training days; sleep rating ↑ on pre-travel vs. match days; nº of awakenings ↑ on pre-travel, training days and matches vs. travel days; jet lag ↑ match vs. pre-travel; sleep ↓ pre-travel, travel and match vs. post-travel; NS for ASSQ	LDTT ↓ sleep vs. pre-travel and tournament days; travel and competition disrupted routines and sleep
Biggins et al. (2022) [80]	Observational, longitudinal, repeated-measures	elite soccer athletes (*n* = 41)	Soccer	Ireland to Taiwan (eastward, 7 time zones)	n/a	Jet lag ↑ pre-competition vs. competitions; Symptoms lasted up to ≤10 d on females, ≤13 d on males; no association was found between chronotype and jet lag; 76% were intermediate type	Jet lag may persist; young athletes cope better; sleep hygiene is insufficient; females ↑ pre-sleep anxiety; eastward 7 time zones ⟶ ↓ sleep, ↓ quality, up to ≤13 d
Charest et al. (2022) [81]	Longitudinal Observational Study	All NHL teams from seasons 2013–2020	Ice Hockey	n/a	n/a	NS associations between circadian change or travel distance and most hockey performance outcomes; GameDistance was strongly associated with GoalDifferential when controlling for TZ, but not for AdjTZ_B; quadratic AdjTZ_B was associated with GoalDifferential when controlling for GameDistance; Interections were observed between GameDistance and circadian change; GameDistance was also associated with DiffActExpFSv%	↑ Distance and circadian change ⟶ ↓ skill performance; +900 km ⟶ −0.04 GoalDifferential; ability/location > travel; circadian misalignment + long travel = worst impact
Glinski et al. (2022) [13]	Longitudinal observational Study	NBA Players from season 1999 to 2000	Basketball	n/a	n/a	Jet lag ⟶ ↓teams FT%; main shooters NS; others ↓; effect only on eastward travel	Jet lag ↓ FT; Eastward travel worsens FT; performance declines are mainly due to circadian disruption; repeated exposure mitigates effects
Jasper et al. (2022) [82]	Original research	elite and professional athletes; general populations; military personnel; occupational shift workers	Athletics	n/a	n/a	Time zone changes ⟶ ↓medal; eastward travel is worse for gold, silver, bronze and total medals	↑TZ ⟶ ↓ performance; eastward travel is worse; ↑ TZs ⟶ larger decline; amateurs and unsupported teams are more affected
Leota et al. (2022) [83]	Longitudinal Observational Study	NBA teams from season 2011/2012 to the 2020/ 2021	Basketball	n/a	n/a	Eastward jet lag ⟶ ↓ wins, points, rebounds, FG%; westward jet lag = no effect on wins, points, rebounds, FG%; + eastward jet lag ⟶ ↓worse points	Eastward jet lag ⟶ ↓ shooting, rebounds, points, wins (home only); westward jet lag no effect; circadian disruption from eastward travel harms performance
Read et al. (2022) [84]	Descriptive longitudinal design	NRL teams from seasons 2007–2019	Rugby	n/a	n/a	Per 1.000 km ⟶ −2.7% win, −1.1 pts; away team Odds Ratio 0.5, −6 pts; +1 day turnaround ⟶ Odds Ratio 0.98, −0.1 pts	Travel ↓ performance; travel impact has been reduced over time (fatigue management, recovery strategies); no inter-state differences
Rossiter et al. (2022) [85]	Observational study	Olympic team support staff (*n* = 9)	n/a	Ireland to Japan (eastward, 8 time zones)	n/a	Jet lag ↑ am day 1 to day 6, pm day 1 to day 4 and day 6; appetite/bowel disturbances on days 1, 3, 5 and 6; cortisol am was ↓ 66% on day 1 to day 5, returns to baseline on day 6; cortisol pm = baseline; sAA ↑ evening on day 3 only; mood am: confusion ↓, depression ↓, vigor ↑; mood pm: confusion ↑, depression ↑, fatigue ↑, vigor ↓; tension ↓ am and pm	Eastward travel crossing 8 TZ ⟶ manage first 7 days for jet lag/fatigue; sleep and mood strategies recommended on pre/post LH travel for Olympic staff
Cullen et al. (2023) [86]	Observational study	referees *n* = 65; (47 males and 18 females; mean age 35 ± 5 years; height 180.3 ± 8.6 cm, weight 79.1 ± 10.5 kgs; BMI 24.2 ± 1.8)	Basketball	n/a	n/a	Poor sleep: early wake (16.4%), jet lag (14.9%), late/evening game (14.9%); jet lag-related poor sleep occurred in the 1st half of the tournament, with 70% on the 1st day	Jet lag ⟶ acute early sleep disruption (70% day 1, 3 days post-flight); earlier bedtimes on day 1 to day 2; sleep likely worse immediately post-travel; jet lag main early factor
Doherty et al. (2023) [87]	Case report	elite international track cycling squad (*n* = 7; *n* = 3 males and *n* = 4 females)	cymincling	Maiorca to Hong Kong (eastward, 7 time zones)	n/a	TIB ↓ 213 min on travel day and ↓ 257 min on arrival; TST ↓ 140 min 4 days before travel, 268 min on travel day, 197 min on arrival, ↑ 87 min on 4th day of arrival; SOL ↑ on travel day; SE ↓ 22.63% o travel day, 13.12% on arrival, ↑ 10.27% on 4th of arrival; effects were observed for TIB × day, TST × day, SE × day and fatigue at bedtime × day	LHT ↓ TIB, TST, SE; LHT ↑ fatigue; jet lag impacts 48 h; travel 5–6 days pre competition; individualized sleep, jet lag strategies are recommended; early post-travel sleep loss harms health and performance
Clements et al. (2023) [88]	Case-study	male senior Australian national football (soccer) team (*n* = 58)	Soccer	n/a	n/a	Trips: ≤3 h TZ %, >3 h 34%, ≥8 h 17%; westward direction 50%, eastward 43%, none 7%; travel ≥ 10 h 51%, ≥24 h 8%; flight ≥ 10 h 41%, ≥20 h 7%; overnight 64% none, 33% one night, 3% two nights; arrival at evenings 39%, early morning 23%, day (09:00–18:00) 39%; location ⟶ significant TZ change, travel and flight time	Most trips are unlikely to impair performance or wellbeing; travel strategies ⟶ location-specific; interventions: sleep, naps, schedule; European based face greater challenges arriving at camp, Australian based players are at higher risk post-return
Clements et al. (2023) [89]	Observational Study	professional footballers (*n* = 68)	Soccer	n/a	n/a	Asia ⟶ Europe and Europe ⟶ Australia ⟶ highest jet lag; Australia ⟶ Asia < Asia ⟶ Asia; transition travel ↑ jet lag; older players ↑ jet lag, experience players ↓; flight path ⟶ wellness, fatigue, sleep, soreness (Europe ⟶ Australia, Asia ⟶ Europe worst); return travel is worse; night arrivals ↑ stress	Jet lag ratings > wellness for travel effects; older players ↑ jet lag, experience players ↓; post-national team return ⟶ recovery challenge; individualized travel, recovery strategies; midday-late afternoon arrival preferred
Clements et al. (2023) [90]	Observational Study	elite senior male national footballers (*n* = 62)	Soccer	n/a	n/a	TZ difference ⟶ wellness, fatigue, sleep, soreness; direction ⟶ sleep, stress; ≥9 h ⟶ worse wellness, fatigue, sleep, improves on day 1 and day 2; soreness was worse after 6–9 h compared with <3 h; stress ↑ after ≥9 h on day 2 compared with 3–6 h	Large TZ changes ⟶ ↑ fatigue, soreness ↓ sleep, wellness; eastward ⟶ worse sleep, ↑ stress; <6 h minimal effect; first 48 h post eastward travel ⟶ sleep interventions
Garbellotto et al. (2023) [91]	Observational design	elite mountain bikers of the French national team [(5 men (age: 28.2 ± 4.5 years; height: 178.6 ± 4.1 cm; body mass: 66.5 6 3.6 kg) 1 woman (age: 19 years; height: 166 cm; mass: 56.1 kg)]	Mouintain Bike	France to Japan (eastward, 8 time zones)	Melatonin; light therapy	Sleep metrics (TST, TIB, WASO; REM) = baseline; SOL ↑ day11; bedtime and wake were earlier on days 9–11; Body temperature recovered to baseline in 3 days; Wingate and ME5 tests showed no differences in Pmean, Pmax, Pmin, fatigue index or cardiorespiratory parameters (VO_2_, VE, RER, HR, VE/VO_2_) at different times of day or after phase adavances/adjustments	3 h sleep–wake advance + melatonin + morning light ⟶ minimized eastward travel disruption; anaerobic/aerobic performance maintained; partial circadian resynchronization pre-arrival; high melatonin ⟶ initial sleep; nonstop flight and early sunrise ⟶ faster body temperature adjustment; peak power, fatiguer ⟶ unchanged
Paule-Koba et al. (2024) [92]	Qualitative	NCAA Division I football and hockey players from the Big Ten, Big 12, WCHA, and Hockey East conferences (*n* = 133)	Hockey; Football	n/a	n/a	Travel enjoyed for new places; away games ⟶ long travel, missed classes, routine disruption; 52% report ↓ athletic performance; legs and sleep affected; academic impact mixed; less time with non-team individuals	Circadian disruption ⟶ jet lag, fatigue; early classes post travel ⟶ ↓ academic performance; reduced travel supports university engagement and overall development
Anderson et al. (2024) [93]	Invited commentary	Visually Impaired Paralympic Athletes	n/a	n/a	n/a	n/a	Paralympic athletes pre, intra, post-flight strategies: bedtime shifting, light exposure, sleep hygiene and melatonin; visual impaired athletes: non-photic cues (meals, exercise, social), maintain habitual sleep–wake; some residual light sensitivity; melatonin; non-photic cues < light circadian entrainment
Varesco et al. (2024) [94]	prospective observational design with non- invasive procedures	elite short-track speed skaters from the Canadian National Team (11 females, age: 24 ± 4 years; height: 169 ± 0.06 m; body mass: 64.4 ± 7.3 kg);	speed skaters	Montréal to Asia (eastward, 10 time zones)	n/a	Sleep ↑ SIA > Baseline, travel, race; Travel, Race ↓ vs. Baseline; SIA +9 min/night, breakpoint on day 5; SE ↑ SIA, slight ↓ over time: bedtime, wake later Race, ↑ linearly SIA; MESOR ↓; CMJ ↑ SIA, Race; RPE ↑ baseline	Sleep debt, jet lag resolved ≤5 days; ensure sleep and activity as zeitgeber; informs long-haul travel planning for coaches, staff
Tsukahara et al. (2024) [9]	Observational Study	athletes (26 men and 20 women) who competed in the 2018 World U20 Athletics Championships	athletics	Tokyo to Helsinki (westward, 7 time zones)	n/a	Performance and agility = AD vs. GD; flexibility ↓ GD; sleep onset ↑, quality ↓, duration ↓, awakenings ↓ GD; hunger, fullness ↓ GD; travel experience ↑ sleep, alertness; males ↑ awakenings; sophomores ↑ sleep difficulty	Prior international travel ↑ adaptation; sophomores ↑ sleep difficulty 4 days post-travel; travel ⟶ digestive circadian shift, ↓ flexibility and sleep quality; scheduling critical; educate young athletes to manage jet lag for performance
Özdalyan et al. (2024) [95]	Case report	NBA Teams from 2000 to 2001 season to 2020–2021 season	Basketball	n/a	n/a	Home win 59.3%, away 40.7%; LB < P and FGM groups; LA > LB, ↑FGA, ↓ W/L and FG%; LS PD/FGM > LA; OA > OS; OA TR > OB	Forward westward circadian shift ↑ performance; backward eastward ↓ performance; full local adaptation ⟶ best away performance; home eastward shift ⟶ ↓ performance; away circadian rhythm shift negative, forward worse; consider in game planning
Rossiter et al. (2024) [96]	Original research	Irish HP athletes, coaches, and support staff, >18 years and competing or working with athletes at an elite level (professional, Olympic/Paralympic, international, and national); *n* = 144	Athletics; Boxing; clay target shooting; cricket; cycling; diving; golf; gymnastics; hockey; rugby; sailing; skeleton; swimming; triathlon	n/a	n/a	LH travel ⟶ ↓ physical (86.4%) and mental (72.7%) performance, ↑ illness, injury risk (86.4%), 93.2% symptoms ≤ 3 days, fatigue most common; females ↑ fatigue and appetite; age, chronotype, experience, TZ affect symptoms; recovery ≤ 7 fays, longer with more TZs; travel strategies widely used (sleep, meals, movement, stretching, fluids); ≥9 TZs ⟶ sleep schedule adjusted in-flight	More TZs ⟶ longer recovery; all recovered ≤7 d (most ≤ 3 d); early waking, fatigue ↑ with TZs; ≤3 TZ also experienced sleep, fatigue issues; strategies; schedule, naps, sleep hygiene, movement; females and evening chronotypes ↑ symptoms; athletes, younger↑ sleep adjustment

⟶ (leads to); ↓ (decreased); ↑ (increased); n/a (not applicable); < (less than); > (greater than); ≤ (less than or equal to); ≥ (greater than or equal to).

**Table 3:** Joanna Briggs Institute (JBI) Checklist.

Reference	Type of Study	Q1	Q2	Q3	Q4	Q5	Q6	Q7	Q8	Q9	Q10	Q11	Q12	Q13	RoB (%)	Evaluation	Tool *
Winget et al. (1985) [15]	Narrative Review	Yes	No	No	No	No	Yes	No							29	Include	4
Loat et al. (1989) [3]	Narrative Review	Yes	No	No	Yes	No	Yes	No							43	Include	4
Hill et al. (1993) [25]	Narrative Review	Yes	No	No	No	No	No	No							14	Include	4
Eichner (1994) [18]	Narrative Review	Yes	Yes	No	No	No	No	No							29	Include	4
Reilly et al. (1997) [4]	Narrative Review	No	No	No	No	No	Yes	No							14	Include	4
Waterhouse et al. (2004) [8]	Narrative Review	Yes	No	No	No	No	Yes	No							29	Include	4
Postolache et al. (2005) [30]	Narrative Review	No	No	No	No	No	Yes	No							14	Include	4
Reilly et al. (2005) [10]	Critical Review	Yes	No	No	No	No	Yes	No	No	No	Yes	Yes			36	Include	4
Armstrong (2006) [31]	Critical Review	Yes	No	No	No	No	No	No	No	No	No	Yes			18	Include	4
Reilly et al. (2007) [34]	Narrative Review	Yes	No	No	No	No	Yes								33	Include	4
Meijer et al. (2008) [35]	Narrative Review	Yes	Yes	Yes	No	Yes	Yes								83	Include	4
Pipe (2011) [7]	Narrative Review	Yes	No	No	No	No	No								16	Include	4
Samuels (2012) [6]	Narrative Review	Yes	No	No	No	No	No								16	Include	4
Lee et al. (2012) [14]	Narrative Review	Yes	No	Yes	Yes	No	Yes								66	Include	4
Forbes-Robertson et al. (2012) [12]	Narrative Review	Yes	No	No	No	No	Yes								33	Include	4
Leatherwood et al. (2013) [39]	Narrative Review	Yes	No	No	No	No	Yes								33	Include	4
Stellingwerff et al. (2014) [41]	Narrative Review	No	No	No	No	No	Yes								16	Include	4
Simmons et al. (2015) [44]	Narrative Review	No	No	No	No	No	Yes								16	Include	4
Williams et al. (2017) [49]	Narrative Review	Yes	No	No	No	No	Yes								33	Include	4
Silva et al. (2019) [56]	Narrative Review	Yes	No	No	No	No	Yes								33	Include	4
Halson et al. (2019) [57]	Narrative Review	Yes	No	No	No	No	Yes								33	Include	4
Zubac et al. (2020) [66]	Narrative Review	Yes	No	No	No	No	Yes								33	Include	4
Janse van Rensburg et al. (2021) [71]	Narrative Review	Yes	Yes	No	Yes	No	Yes								66	Include	4
Janse van Rensburg et al. (2020) [65]	Systematic Review	Yes	Yes	Yes	No	Yes	Yes	Yes	Yes	Yes	Yes	Yes			91	Include	4
Rossiter et al. (2022) [77]	Systematic Review	Yes	No	No	Yes	No	Yes	Yes	Yes	No	Yes	Yes			64	Include	4
GLOBAL RISK OF BIAS SCORE (%)															36		
Jehue et al. (1993) [17]	Observational	Yes	No	No	Yes	No	Yes	Yes	Yes	No	No	Yes			55	Include	5
Smith et al. (1997) [16]	Observational	Yes	Yes	Yes	No	No	Yes	Yes	Yes	Yes	No	Yes			73	Include	5
Waterhouse (2002) [2]	Observational	No	No	No	Yes	Yes	Yes	No	Yes	No	No	Yes			45	Include	5
Lemmer et al. (2002) [29]	Observational	Yes	No	No	Yes	No	Yes	Yes	Yes	Yes	No	Yes			64	Include	5
Bullock et al. (2007) [33]	Observational	Yes	Yes	Yes	No	No	Yes	Yes	Yes	Yes	No	Yes			73	Include	5
Fowler et al. (2015) [43]	Observational	Yes	Yes	Yes	No	No	Yes	Yes	Yes	Yes	No	Yes			73	Include	5
Fowler et al. (2016) [46]	Observational	Yes	Yes	Yes	Yes	Yes	Yes	Yes	Yes	Yes	No	Yes			91	Include	5
Fullagar et al. (2016) [47]	Observational	Yes	Yes	Yes	Yes	No	Yes	Yes	Yes	Yes	Yes	Yes			91	Include	5
Silva et al. (2016) [45]	Observational	Yes	No	No	No	No	Yes	No	Yes	Yes	No	Yes			45	Include	5
Fowler et al. (2017) [50]	Observational	Yes	Yes	Yes	Yes	Yes	Yes	Yes	Yes	Yes	No	Yes			91	Include	5
Fowler et al. (2017) [48]	Observational	Yes	Yes	Yes	Yes	Yes	Yes	Yes	No	Yes	Yes	Yes			91	Include	5
Kölling et al. (2017) [51]	Observational	Yes	Yes	No	Yes	No	Yes	Yes	Yes	Yes	No	Yes			73	Include	5
Song et al. (2017) [52]	Observational	Yes	Yes	Yes	Yes	Yes	Yes	No	Yes	Yes	Yes	Yes			91	Include	5
Roy et al. (2018) [53]	Observational	Yes	Yes	Yes	Yes	Yes	Yes	Yes	Yes	Yes	Yes	Yes			100	Include	5
Thornton et al. (2018) [55]	Observational	Yes	Yes	Yes	No	No	Yes	Yes	Yes	Yes	No	Yes			73	Include	5
Lo et al. (2019) [60]	Observational	Yes	Yes	Yes	Yes	No	Yes	Yes	Yes	Yes	No	Yes			82	Include	5
Fullagar et al. (2019) [59]	Observational	Yes	Yes	Yes	Yes	Yes	Yes	Yes	Yes	Yes	No	Yes			91	Include	5
Lo et al. (2019) [62]	Observational	Yes	Yes	Yes	Yes	No	Yes	Yes	Yes	Yes	No	Yes			82	Include	5
Augusto et al. (2021) [68]	Observational	Yes	Yes	Yes	No	No	Yes	Yes	Yes	Yes	No	Yes			73	Include	5
Leduc et al. (2021) [73]	Observational	Yes	Yes	Yes	Yes	No	Yes	Yes	No	Yes	No	Yes			73	Include	5
Lalor et al. (2021) [72]	Observational	Yes	Yes	Yes	Yes	No	Yes	Yes	Yes	Yes	No	Yes			82	Include	5
Charest et al. (2021) [69]	Observational	Yes	Yes	No	Yes	No	Yes	Yes	Yes	Yes	No	Yes			73	Include	5
Smithies et al. (2021) [75]	Observational	Yes	Yes	Yes	No	No	Yes	Yes	Yes	Yes	No	Yes			73	Include	5
Charest et al. (2022) [81]	Observational	Yes	Yes	Yes	Yes	Yes	Yes	Yes	Yes	Yes	No	Yes			91	Include	5
Glinski et al. (2022) [13]	Observational	Yes	Yes	Yes	No	No	Yes	Yes	Yes	Yes	No	Yes			73	Include	5
Leota et al. (2022) [83]	Observational	Yes	Yes	Yes	No	No	Yes	Yes	Yes	Yes	No	Yes			73	Include	5
Flatt et al. (2022) [78]	Observational	Yes	Yes	Yes	Yes	Yes	Yes	Yes	Yes	Yes	No	Yes			91	Include	5
Read et al. (2022) [84]	Observational	Yes	Yes	Yes	No	No	Yes	Yes	Yes	Yes	No	Yes			73	Include	5
Biggins et al. (2022) [80]	Observational	Yes	Yes	Yes	No	No	Yes	Yes	Yes	Yes	No	Yes			73	Include	5
Everett et al. (2022) [76]	Observational	Yes	Yes	Yes	No	No	Yes	No	Yes	Yes	No	Yes			64	Include	5
Rossiter et al. (2022) [77]	Observational	Yes	Yes	Yes	No	No	Yes	Yes	Yes	Yes	No	Yes			73	Include	5
Clements et al. (2023) [89]	Observational	Yes	Yes	No	No	No	Yes	Yes	Yes	Yes	No	Yes			64	Include	5
Clements et al. (2023) [90]	Observational	Yes	No	No	No	No	Yes	Yes	Yes	Yes	No	Yes			55	Include	5
Cullen et al. (2023) [86]	Observational	Yes	Yes	No	No	No	Yes	Yes	Yes	Yes	No	Yes			64	Include	5
Garbellotto et al. (2023) [91]	Observational	Yes	Yes	Yes	No	No	Yes	Yes	Yes	Yes	No	Yes			73	Include	5
Tsukahara et al. (2024) [9]	Observational	Yes	Yes	No	No	No	Yes	No	Yes	Yes	No	No			45	Include	5
Varesco et al. (2024) [94]	Observational	Yes	Yes	Yes	No	No	Yes	Yes	Yes	Yes	No	Yes			73	Include	5
GLOBAL RISK OF BIAS SCORE (%)															74		
Fuller et al. (2015) [42]	Cohort study	Yes	Yes	No	No	No	Yes	Yes	Yes	Yes	No	Yes			64	Include	5
Stevens et al. (2018) [54]	Cohort study	Yes	Yes	Yes	No	No	Yes	Yes	Yes	Yes	No	Yes			73	Include	5
Jasper et al. (2022) [82]	Cohort study	No	No	No	No	No	Yes	No	Yes	Yes	No	Yes			36	Include	5
GLOBAL RISK OF BIAS SCORE (%)															58		
Milne et al. (2007) [32]	Case report	No	Yes	No	Yes	No	No	No	Yes						38	Include	2
GLOBAL RISK OF BIAS SCORE (%)															38		
Cardinali et al. (2002) [28]	Case study	Yes	Yes	No	Yes	Yes	Yes	No	Yes	No	Yes				70	Include	1
Montaruli et al. (2009) [37]	Case study	No	Yes	Yes	No	No	No	No	Yes	No	Yes				40	Include	1
Lastella, M. et al. (2019) [63]	Case study	No	Yes	No	No	No	Yes	No	Yes	No	Yes				40	Include	1
Lever, J. R. et al. (2022) [79]	Case study	No	No	No	No	No	No	No	Yes	No	Yes				20	Include	1
Doherty, R. et al. (2023) [87]	Case study	No	No	No	No	No	Yes	Yes	Yes	No	Yes				40	Include	1
Clements, E. et al. (2023) [88]	Case study	Yes	No	No	No	No	Yes	Yes	Yes	Yes	Yes				60	Include	1
Özdalyan, F. et al. (2024) [95]	Case study	Yes	Yes	Yes	Yes	Yes	No	No	Yes	Yes	Yes				80	Include	1
GLOBAL RISK OF BIAS SCORE (%)															50		
Manfredini, R. et al. (2000) [26]	Quasi-experimental	Yes	No	No	Yes	Yes	No	No	Yes	Yes					56	Include	3
Chapman, D. W. et al. (2012) [38]	Quasi-experimental	Yes	Yes	No	No	Yes	Yes	Yes	Yes	Yes					78	Include	3
Broatch, J. R. et al. (2019) [58]	Quasi-experimental	Yes	Yes	Yes	No	Yes	Yes	Yes	Yes	Yes					89	Include	3
Atalag, O. et al. (2019) [61]	Quasi-experimental	Yes	Yes	No	No	Yes	Yes	Yes	Yes	Yes					78	Include	3
Straub, W. F. et al. (2001) [27]	Experimental	Yes	No	Yes	Yes	Yes	Yes	Yes	Yes	Yes					89	Include	3
Dranitsin, O. V. et al. (2008) [36]	Experimental	Yes	No	Yes	Yes	Yes	Yes	No	Yes	Yes					78	Include	3
Hoshikawa, M. et al. (2020) [97]	Experimental	Yes	Yes	Yes	No	Yes	Yes	Yes	Yes	Yes					89	Include	3
GLOBAL RISK OF BIAS SCORE (%)															80		
Thompson, A. et al. (2013) [40]	Parallel-group RCT	Yes	Yes	Yes	No	No	Yes	No	Yes	Yes	Yes	Yes	Yes	Yes	77	Include	6
Fowler, P. M. et al. (2021) [70]	RCT	No	No	Yes	No	No	Yes	No	Yes	Yes	Yes	Yes	Yes	Yes	62	Include	6
GLOBAL RISK OF BIAS SCORE (%)															70		
Lo, M. et al. (2021) [74]	Qualitative description	Yes	Yes	Yes	Yes	Yes	Yes	Yes	No	Yes	Yes				90	Include	7
Paule-Koba, A. L. et al. (2024) [92]	Qualitative description	Yes	Yes	Yes	Yes	Yes	Yes	Yes	Yes	Yes	Yes				100	Include	7
GLOBAL RISK OF BIAS SCORE (%)															95		
Schobersberger, W. et al. (2012) [5]	Commentary	Yes	No	No	Yes	Yes	Yes								67	Include	8
Nikolaidi, M. K. et al. (2021) [67]	Commentary	Yes	Yes	Yes	Yes	Yes	Yes								100	Include	8
Anderson, T. et al. (2024) [93]	Commentary	No	Yes	Yes	Yes	Yes	No								67	Include	8
Reilly, T. et al. (2007) [11]	Position statement	Yes	Yes	Yes	Yes	Yes	Yes								100	Include	8
GLOBAL RISK OF BIAS SCORE (%)															84		
Rossiter, A. et al. (2024) [96]	Cross-sectional survey	Yes	No	No	No	No	No	No	Yes						25	Include	9
GLOBAL RISK OF BIAS SCORE (%)															25		

* 1. Checklist JBI for case series; 2. Checklist JBI for case reports; 3. Checklist JBI for Quasi-experimental studies; 4. Checklist JBI for systematic reviews and research syntheses; 5. Checklist JBI for cohort studies; 6. Checklist JBI for Randomized Controlled Trials; 7. Checklist JBI for qualitative research; 8. Checklist JBI for expert opinion; 9. Checklist JBI for analytical cross sectional studies. “Yes” (low risk of bias); “No” No (high risk of bias); Source: https://jbi.global/critical-appraisal-tools (accessed on 3 December 2024).

## Data Availability

No new data were created or analyzed in this study. Data sharing is not applicable to this article.

## Supplementary Materials

The following supporting information can be downloaded at: https://www.mdpi.com/article/10.3390/sports14030093/s1, File S1: PRISMA 2020 checklist.

## Abbreviations

AM	Ante Meridiem
ATP	Adenosine Triphosphate
ASSQ	Athlete Sleep Screening Questionnaire
BDJ	Broad Jump
BFR	Blood Flow Restriction
CG	Control Group
CMJ	Countermovement Jump
CV	Cardiovascular
EC	East Coast
ES	Effect Size
GS	Grip Strength
HR	Heart Rate
HRV	Heart Rate Variability
INT	Intervention Group
JBI	Joanna Briggs Institute
LCMJ	Loaded Countermovement Jump
LH	Long-Haul
MBB	Men’s Basketball
ME5	5-Minute Maximal Effort Test
MLB	Major League Baseball
MNF	Monday Night Football
NBA	National Basketball Association
NCAA	National Collegiate Athletic Association
NHL	National Hockey League
NOS	Newcastle-Ottawa Scale
NRL	National Rugby League
NS	Not Significant
PICOS	Participants, Interventions, Comparators, Outcomes, Study Design
PRE	Pre-Intervention
PPT	Peak Power Test
PROSPERO	International Prospective Register of Systematic Reviews
PRISMA	Preferred Reporting Items for Systematic Reviews and Meta-Analyses
RPE	Rating of Perceived Exertion
REM	Rapid Eye Movement sleep
ROC	Receiver Operating Characteristic
SD	Standard Deviation
SE	Sleep Efficiency
SJ	Squat Jump
SOL	Sleep Onset Latency
SSC	Stretch-Shortening Cycle
S&C	Strength and Conditioning
SM	Supplemented
SCN	Suprachiasmatic Nucleus
TIB	Time in Bed
TST	Total Sleep Time
TZ	Time Zone
UR	Upper Respiratory
$\dot{V}$O_2max_	Maximal Oxygen Uptake
WASO	Wake After Sleep Onset
WC	World Cup
WHR	Waist-Hip Ratio
